# Supplementary material for: Association of hydralazine use with risk of hematologic neoplasms in patients with hypertension: A nationwide population-based cohort study in Taiwan
Source: PLoS Med. 2025 Dec 4;22(12):e1004646. doi: 10.1371/journal.pmed.1004646 (PMC12677456; doi:10.1371/journal.pmed.1004646)
Supplement: S1 File — Table A. Baseline characteristics of the hydralazine cohort compared to patients with hypertension excluded during initial screening. Table B. ICD-9-CM coding and definition. Table C. Comparison of the adjusted subdistribution hazard ratio of hematologic neoplasms according to subgroup stratified by prescription duration of hydralazine in first-event and multiple-event models in a competing risk model. Table D. Multivariable risk regression analysis of hematologic neoplasm development in patients without/with hypertension in competing risk model. Table E. Adjusted hazard ratio for remaining hematologic neoplasm subgroups, stratified by prescription duration of hydralazine. Table F. Leave-one-out analysis for comparison of adjusted hazard ratio of hematologic neoplasms according to subgroup stratified by prescription duration of hydralazine in first-event and multiple-event models in a competing risk model. Table G. Tracking years in patients with hypertension by prescription duration of hydralazine. Table H. Tracking years from initiating hydralazine prescription to having hematologic neoplasms in patients with hypertension. Table I. Endpoint characteristics of patients with hypertension by prescription duration of hydralazine, 2000–2015. Table J. Mortality analysis of patients with hypertension by prescription duration of hydralazine, 2000–2015. Table K. Unadjusted (crude) hazard ratios for risk factors associated with hematologic neoplasm development. Table L. Unadjusted (crude) hazard ratios for hematologic neoplasm development, stratified by prescription duration of hydralazine. Table M. Unadjusted (crude) hazard ratios for sensitivity analysis of hematologic neoplasm development. Table N. Unadjusted (crude) subdistribution hazard ratios for first-event and multiple-event models. Table O. Unadjusted (crude) hazard ratios for hematologic neoplasm risk associated with hypertension. Table P. Unadjusted (crude) subdistribution hazard ratios for leave-one-out sensiti [file pmed.1004646.s002.docx]

| **Table A. Baseline Characteristics of the Hydralazine Cohort Compared to Patients with Hypertension Excluded During Initial Screening** | | | | | | | |
| --- | --- | --- | --- | --- | --- | --- | --- |
| **Variables** |  | **Hydralazine ≥ 180 days** | |  | **Exclusion** | | ***P**** |
|  |  | ***n*** | **%** |  | ***n*** | **%** |  |
| **Total** | | 59,786 |  |  | 1,026 |  |  |
| **Sex** | | | | | | | < .001 |
| Male | | 31,200 | 52.19 |  | 678 | 66.08 |  |
| Female | | 28,586 | 47.81 |  | 348 | 33.92 |  |
| **Age (years)** | | 60.82 ± 13.86 | |  | 52.18± 11.24 | | < .001 |
| **Age group (years)** | | | | | | | < .001 |
| 20-29 | | 0 | 0.00 |  | 5 | 0.49 |  |
| 30-39 | | 441 | 0.74 |  | 53 | 5.17 |  |
| 40-49 | | 3,516 | 5.88 |  | 120 | 11.70 |  |
| 50-59 | | 10,458 | 17.49 |  | 164 | 15.98 |  |
| ≥60 | | 11,065 | 18.51 |  | 313 | 30.51 |  |
| **Insured premium (NTD)** | | | | | | | < .001 |
| <18,000 | | 52,341 | 87.55 |  | 796 | 77.58 |  |
| 18,000-34,999 | | 4,778 | 7.99 |  | 134 | 13.06 |  |
| ≥35,000 | | 2,667 | 4.46 |  | 96 | 9.36 |  |
| **Normal pregnancy** | | 6,475 | 10.83 |  | 100 | 9.75 | .268 |
| **Comorbidities** | | | | | | |  |
| CHF | | 798 | 1.33 |  | 56 | 5.46 | < .001 |
| PE | | 33 | 0.06 |  | 4 | 0.39 | .003 |
| GI hemorrhage | | 120 | 0.20 |  | 2 | 0.19 | .967 |
| Cerebral thrombosis | | 145 | 0.24 |  | 3 | 0.29 | .742 |
| IHD | | 1,014 | 1.70 |  | 25 | 2.44 | .087 |
| Vascular insufficiency of  intestine | | 198 | 0.33 |  | 5 | 0.49 | .401 |
| Obesity | | 67 | 0.11 |  | 1 | 0.10 | .890 |
| Malignant neoplasm of  kidney/renal pelvis | | 1,978 | 3.31 |  | 46 | 4.48 | .043 |
| Acute glomerulonephritis/  Nephrotic syndrome | | 484 | 0.81 |  | 24 | 2.34 | < .001 |
| Proteinuria | | 333 | 0.56 |  | 6 | 0.58 | .831 |
| Gestational hypertension | | 482 | 0.81 |  | 9 | 0.88 | .724 |
| Asthma | | 3,327 | 5.56 |  | 66 | 6.43 | .230 |
| CCI_R | | 0.82 ± 1.22 | |  | 1.05 ± 1.31 | | < .001 |
| **Medications** | |  | |  |  | |  |
| Aspirin | | 8,976 | 15.01 |  | 137 | 13.35 | .139 |
| Celecoxib | | 7,378 | 12.34 |  | 120 | 11.70 | .533 |
| Itraconazole | | 2,885 | 4.83 |  | 33 | 3.22 | .015 |
| Mebendazole | | 8,125 | 13.59 |  | 68 | 6.63 | < .001 |
| Leflunomide | | 3,876 | 6.48 |  | 79 | 7.70 | .117 |
| Thalidomide | | 6,022 | 10.07 |  | 106 | 10.33 | .785 |
| Valproate | | 5,227 | 8.74 |  | 48 | 4.68 | < .001 |
| Metformin | | 9,896 | 16.55 |  | 191 | 18.62 | .083 |
| Auranofin | | 3,542 | 5.92 |  | 96 | 9.36 | < .001 |
| Statins | | 7,896 | 13.21 |  | 134 | 13.06 | .891 |
| Bisphosphonates | | 5,014 | 8.39 |  | 86 | 8.38 | .996 |
| Bromocriptine | | 6,156 | 10.30 |  | 124 | 12.09 | .062 |
| Chlorprothixene | | 7,013 | 11.73 |  | 101 | 9.84 | .062 |
| Clotrimazole | | 5,882 | 9.84 |  | 98 | 9.55 | .760 |
| Quinacrine | | 4,782 | 8.00 |  | 73 | 7.12 | .301 |
| Ivermectin | | 5,079 | 8.50 |  | 84 | 8.19 | .725 |
| Verteporfin | | 3,846 | 6.43 |  | 61 | 5.95 | .528 |
| Clarithromycin | | 2,115 | 3.54 |  | 35 | 3.41 | .932 |
| Hydroxychloroquine | | 5,357 | 8.96 |  | 79 | 7.70 | .161 |
| Tofacitinib | | 6,014 | 10.06 |  | 111 | 10.82 | .423 |
| Gefitinib | | 5,511 | 9.22 |  | 104 | 10.14 | .314 |
| Curcumin | | 4,056 | 6.78 |  | 66 | 6.43 | .657 |
| Chlorhexidine | | 3,798 | 6.35 |  | 58 | 5.65 | .401 |
| Axitinib | | 2,458 | 4.11 |  | 34 | 3.31 | .233 |
| **Season of index date** | | | | | | | .211 |
| Spring (Mar-May) | | 14,898 | 24.92 |  | 252 | 24.56 |  |
| Summer (Jun-Aug) | | 15,207 | 25.44 |  | 267 | 26.02 |  |
| Autumn (Sep-Nov) | | 13,782 | 23.05 |  | 259 | 25.24 |  |
| Winter (Dec-Feb) | | 15,889 | 26.59 |  | 248 | 24.17 |  |
| **Location** | | | | | | | < .001 |
| Northern Taiwan | | 22,518 | 37.66 |  | 392 | 38.21 |  |
| Middle Taiwan | | 17,184 | 28.74 |  | 233 | 22.71 |  |
| Southern Taiwan | | 11,297 | 18.90 |  | 212 | 20.66 |  |
| Eastern Taiwan | | 7,022 | 11.75 |  | 153 | 14.91 |  |
| Outlets islands | | 1,765 | 2.95 |  | 36 | 3.51 |  |
| **Urbanization level** | | | | | | | .001 |
| 1 (The highest) | | 21,449 | 35.88 |  | 350 | 34.11 |  |
| 2 | | 19,780 | 33.08 |  | 297 | 28.95 |  |
| 3 | | 8,245 | 13.79 |  | 168 | 16.37 |  |
| 4 (The lowest) | | 10,312 | 17.25 |  | 211 | 20.57 |  |
| **Levels of hospitals** | | | | | | | .007 |
| Medical center | | 20,745 | 34.70 |  | 310 | 30.21 |  |
| Regional hospital | | 20,110 | 33.64 |  | 382 | 37.23 |  |
| Local hospital | | 18,931 | 31.66 |  | 334 | 32.55 |  |
| Abbreviations: NTD = New Taiwan dollar; CHF = congestive heart failure; PE = pulmonary embolism; GI = gastrointestinal; IHD = ischemic heart disease; CCI_R = Charlson Comorbidity Index_Revised.  **P*: Chi-square test was used for all categorical variables, whereas the *t* test was used for continuous variables. | | | | | | | |

| **Table B. ICD-9-CM coding and Definition** | | | | | | | | | | | | | | | | | | | | | | | | |  |  |  |  |
| --- | --- | --- | --- | --- | --- | --- | --- | --- | --- | --- | --- | --- | --- | --- | --- | --- | --- | --- | --- | --- | --- | --- | --- | --- | --- | --- | --- | --- |
|  | | | | **ICD-9-CM coding/ Definition** | | | | | | | | | | | | | | | | | | | | |  |  |  |  |
| **Study population:** | | | |  | | | | | | | | | | | | | | | | | | | | |  |  |  |  |
| Hypertension | | | | 401-405 | | | | | | | | | | | | | | | | | | | | |  |  |  |  |
| Using Antihypertensive agents | | | | Any of below continuous use ≥180 days | | | | | | | | | | | | | | | | | | | | |  |  |  |  |
| Exposure Group | | | | Hydralazine ≥180 days | | | | | | | | | | | | | | | | | | | | |  |  |  |  |
| Reference Group | | | | Hydralazine <180 days | | | | | | | | | | | | | | | | | | | | |  |  |  |  |
| Benazepril hydrochloride | | | | Continuous use ≥180 days | | | | | | | | | | | | | | | | | | | | |  |  |  |  |
| Captopril | | | | Continuous use ≥180 days | | | | | | | | | | | | | | | | | | | | |  |  |  |  |
| Enalapril maleate | | | | Continuous use ≥180 days | | | | | | | | | | | | | | | | | | | | |  |  |  |  |
| Fosinopril sodium | | | | Continuous use ≥180 days | | | | | | | | | | | | | | | | | | | | |  |  |  |  |
| Lisinopril | | | | Continuous use ≥180 days | | | | | | | | | | | | | | | | | | | | |  |  |  |  |
| Perindopril | | | | Continuous use ≥180 days | | | | | | | | | | | | | | | | | | | | |  |  |  |  |
| Quinapril hydrochloride | | | | Continuous use ≥180 days | | | | | | | | | | | | | | | | | | | | |  |  |  |  |
| Ramipril | | | | Continuous use ≥180 days | | | | | | | | | | | | | | | | | | | | |  |  |  |  |
| Amlodipine besylate | | | | Continuous use ≥180 days | | | | | | | | | | | | | | | | | | | | |  |  |  |  |
| Diltiazem hydrochloride | | | | Continuous use ≥180 days | | | | | | | | | | | | | | | | | | | | |  |  |  |  |
| Felodipine | | | | Continuous use ≥180 days | | | | | | | | | | | | | | | | | | | | |  |  |  |  |
| Nicardipine | | | | Continuous use ≥180 days | | | | | | | | | | | | | | | | | | | | |  |  |  |  |
| Nifedipine | | | | Continuous use ≥180 days | | | | | | | | | | | | | | | | | | | | |  |  |  |  |
| Verapamil hydrochloride | | | | Continuous use ≥180 days | | | | | | | | | | | | | | | | | | | | |  |  |  |  |
| Chlorthalidone | | | | Continuous use ≥180 days | | | | | | | | | | | | | | | | | | | | |  |  |  |  |
| Chlorothiazide | | | | Continuous use ≥180 days | | | | | | | | | | | | | | | | | | | | |  |  |  |  |
| Hydrochlorothiazide | | | | Continuous use ≥180 days | | | | | | | | | | | | | | | | | | | | |  |  |  |  |
| Indapamide | | | | Continuous use ≥180 days | | | | | | | | | | | | | | | | | | | | |  |  |  |  |
| Metolazone | | | | Continuous use ≥180 days | | | | | | | | | | | | | | | | | | | | |  |  |  |  |
| Telmisartan | | | | Continuous use ≥180 days | | | | | | | | | | | | | | | | | | | | |  |  |  |  |
| Normal pregnancy | | | | V22 | | | | | | | | | | | | | | | | | | | | |  |  |  |  |
| **Outcomes: Hematologic neoplasms** | | | | Any of the listed below | | | | | | | | | | | | | | | | | | | | |  |  |  |  |
| 1. Lymphosarcoma and reticulosarcoma | | | | 200 | | | | | | | | | | | | | | | | | | | | |  |  |  |  |
| 1. Hodgkin's disease | | | | 201 | | | | | | | | | | | | | | | | | | | | |  |  |  |  |
| 1. Other malignant neoplasms of lymphoid and histiocytic tissue | | | | 202 | | | | | | | | | | | | | | | | | | | | |  |  |  |  |
| 1. Multiple myeloma and immunoproliferative neoplasms | | | | 203 | | | | | | | | | | | | | | | | | | | | |  |  |  |  |
| 1. Lymphoid leukemia | | | | 204 | | | | | | | | | | | | | | | | | | | | |  |  |  |  |
| 1. Myeloid leukemia | | | | 205 | | | | | | | | | | | | | | | | | | | | |  |  |  |  |
| 1. Monocytic leukemia | | | | 206 | | | | | | | | | | | | | | | | | | | | |  |  |  |  |
| 1. Other specified leukemia | | | | 207 | | | | | | | | | | | | | | | | | | | | |  |  |  |  |
| 1. Leukemia of unspecified cell type | | | | 208 | | | | | | | | | | | | | | | | | | | | |  |  |  |  |
| 1. Neoplasm of uncertain behavior | | | | 238.4 (polycythemia vera), 238.5 (neoplasm of uncertain behavior of histiocytic and mast cells), 238.6 (neoplasm of uncertain behavior of plasma cells), 238.71 (essential thrombocythemia), 238.72 (low grade myelodysplastic syndrome lesions), 238.73 (high grade myelodysplastic syndrome lesions), 238.74 (myelodysplastic syndrome with 5q deletion), 238.75 (myelodysplastic syndrome, unspecified), 238.76 (myelofibrosis with myeloid metaplasia), 238.79 (other lymphatic and hematopoietic tissues), 289.83 (myelofibrosis) | | | | | | | | | | | | | | | | | | | | |  |  |  |  |
| 1. MDS | | | | 238.72 (low grade myelodysplastic syndrome lesions), 238.73 (high grade myelodysplastic syndrome lesions), 238.74 (myelodysplastic syndrome with 5q deletion), 238.75 (myelodysplastic syndrome, unspecified) | | | | | | | | | | | | | | | | | | | | |  |  |  |  |
| 1. Paraproteinemia | | | | 273.1 (monoclonal paraproteinemia), 273.2 (other paraproteinemias), 273.3 (macroglobulinemia), 273.8 (other disorders of plasma protein metabolism), 273.9 (unspecified disorder of plasma protein metabolism) | | | | | | | | | | | | | | | | | | | | |  |  |  |  |
| (13) Other polycythemia | | | | 289.0 (polycythemia, secondary), 289.6 (familial polycythemia) | | | | | | | | | | | | | | | | | | | | |  |  |  |  |
| **Comorbidities** | | | | Including preexisting ICD-9-CM diagnosis before index date | | | | | | | | | | | | | | | | | | | | |  |  |  |  |
| Congestive heart failure | | | | 428 | | | | | | | | | | | | | | | | | | | | |  |  |  |  |
| Pulmonary embolism | | | | 415.1 | | | | | | | | | | | | | | | | | | | | |  |  |  |  |
| Gastrointestinal hemorrhage | | | | 578 | | | | | | | | | | | | | | | | | | | | |  |  |  |  |
| Cerebral thrombosis | | | | 434.0 | | | | | | | | | | | | | | | | | | | | |  |  |  |  |
| Ischemic heart disease | | | | 411 | | | | | | | | | | | | | | | | | | | | |  |  |  |  |
| Vascular insufficiency of intestine | | | | 557 | | | | | | | | | | | | | | | | | | | | |  |  |  |  |
| Obesity | | | | 278 | | | | | | | | | | | | | | | | | | | | |  |  |  |  |
| Malignant neoplasm of kidney / renal pelvis | | | | 189.0 (malignant neoplasm of kidney, except pelvis), 189.1(malignant neoplasm of renal pelvis) | | | | | | | | | | | | | | | | | | | | |  |  |  |  |
| Acute glomerulonephritis / Nephrotic syndrome | | | | 580 (acute glomerulonephritis), 581 (nephrotic syndrome) | | | | | | | | | | | | | | | | | | | | |  |  |  |  |
| Proteinuria | | | | 791.0 | | | | | | | | | | | | | | | | | | | | |  |  |  |  |
| Gestational hypertension | | | | 642 | | | | | | | | | | | | | | | | | | | | |  |  |  |  |
| Asthma | | | | 493 | | | | | | | | | | | | | | | | | | | | |  |  |  |  |
| Charlson Comorbidity Index_revised | | | | CCI excluded the following diseases, including hematologic neoplasms, hypertension, CHF, cerebral thrombosis, IHD, malignant neoplasm of kidney /renal pelvis, acute glomerulonephritis /nephrotic syndrome, and asthma | | | | | | | | | | | | | | | | | | | | |  |  |  |  |
| **Medications** | | | | Including prescription before index date | | | | | | | | | | | | | | | | | | | | |  |  |  |  |
| Aspirin | | | | Continuous use ≥180 days | | | | | | | | | | | | | | | | | | | | |  |  |  |  |
| Celecoxib | | | | Continuous use ≥180 days | | | | | | | | | | | | | | | | | | | | |  |  |  |  |
| Itraconazole | | | | Continuous use ≥180 days | | | | | | | | | | | | | | | | | | | | |  |  |  |  |
| Mebendazole | | | | Continuous use ≥180 days | | | | | | | | | | | | | | | | | | | | |  |  |  |  |
| Leflunomide | | | | Continuous use ≥180 days | | | | | | | | | | | | | | | | | | | | |  |  |  |  |
| Thalidomide | | | | Continuous use ≥180 days | | | | | | | | | | | | | | | | | | | | |  |  |  |  |
| Valproate | | | | Continuous use ≥180 days | | | | | | | | | | | | | | | | | | | | |  |  |  |  |
| Metformin | | | | Continuous use ≥180 days | | | | | | | | | | | | | | | | | | | | |  |  |  |  |
| Auranofin | | | | Continuous use ≥180 days | | | | | | | | | | | | | | | | | | | | |  |  |  |  |
| Statins | | | | Continuous use ≥180 days; Included the following medications: nystatin, lovastatin, pravastatin, simvastatin, atorvastatin, pitavastatin, rosuvastatin, cilastatin | | | | | | | | | | | | | | | | | | | | |  |  |  |  |
| Bisphosphonates | | | | Continuous use ≥180 days; Included alendronate and risedronate | | | | | | | | | | | | | | | | | | | | |  |  |  |  |
| Bromocriptine | | | | Continuous use ≥180 days | | | | | | | | | | | | | | | | | | | | |  |  |  |  |
| Chlorprothixene | | | | Continuous use ≥180 days | | | | | | | | | | | | | | | | | | | | |  |  |  |  |
| Clotrimazole | | | | Continuous use ≥180 days | | | | | | | | | | | | | | | | | | | | |  |  |  |  |
| Quinacrine | | | | Continuous use ≥180 days | | | | | | | | | | | | | | | | | | | | |  |  |  |  |
| Ivermectin | | | | Continuous use ≥180 days | | | | | | | | | | | | | | | | | | | | |  |  |  |  |
| Verteporfin | | | | Continuous use ≥180 days | | | | | | | | | | | | | | | | | | | | |  |  |  |  |
| Clarithromycin | | | | Continuous use ≥180 days | | | | | | | | | | | | | | | | | | | | |  |  |  |  |
| Hydroxychloroquine | | | | Continuous use ≥180 days | | | | | | | | | | | | | | | | | | | | |  |  |  |  |
| Tofacitinib | | | | Continuous use ≥180 days | | | | | | | | | | | | | | | | | | | | |  |  |  |  |
| Gefitinib | | | | Continuous use ≥180 days | | | | | | | | | | | | | | | | | | | | |  |  |  |  |
| Curcumin | | | | Continuous use ≥180 days | | | | | | | | | | | | | | | | | | | | |  |  |  |  |
| Chlorhexidine | | | | Continuous use ≥180 days | | | | | | | | | | | | | | | | | | | | |  |  |  |  |
| Axitinib | | | | Continuous use ≥180 days | | | | | | | | | | | | | | | | | | | | |  |  |  |  |
| Abbreviations: ICD-9-CM, International Classification of Diseases, 9th Revision, Clinical Modification; MDS, myelodysplastic syndromes; CCI, Charlson Comorbidity Index; CHF, congestive heart failure; IHD, ischemic heart disease. | | | | | | | | | | | | | | | | | | | | | | | | |  |  |  |  |
| **Table C. Comparison of the Adjusted Subdistribution Hazard Ratio of Hematologic Neoplasms According to Subgroup Stratified by Prescription Duration of Hydralazine in First-Event and Multiple-Event Models in a Competing Risk Model***† | | | | | | | | | | | | | | | | | | | | | | | | | | | |  |
| **Subgroups of Hematologic Neoplasms** | | **Prescription Duration of Hydralazine** | **Population** | **First-Event Model** | | | | | | | | | **Multiple-Event Model** | | | | | | | | | | | | | |  |  |
|  |  |  |  | **Events** | | **Adjusted sHR**§ | | | **95% CI** | | | ***P*** | **Events** | **Adjusted sHR**§ | **95% CI** | | | | | | | ***P*** | | | | |  |  |
| **Overall (Hematologic neoplasms)** | | <180 days | 239,144 | 4,544 | | Reference | | |  | |  |  | - | - | - | | - | | | | | - | | | | |  |  |
|  |  | ≥180 days | 59,786 | 757 | | 0.789 | | | 0.667 | | 0.913 | 2.364E-5 | - | - | - | | - | | | | | - | | | | |  |  |
|  |  | 180-350 days | 19,868 | 294 | | 0.916 | | | 0.767 | | 1.134 | .142 | - | - | - | | - | | | | | - | | | | |  |  |
|  |  | 351-667 days | 19,975 | 245 | | 0.754 | | | 0.618 | | 0.935 | 5.787E-5 | - | - | - | | - | | | | | - | | | | |  |  |
|  |  | ≥668 days | 19,943 | 218 | | 0.666 | | | 0.552 | | 0.831 | 6.004E-6 | - | - | - | | - | | | | | - | | | | |  |  |
| **Lymphosarcoma and reticulosarcoma** | | <180 days | 239,144 | 20 | | Reference | | |  | |  |  | 33 | Reference |  | |  | | | | |  | | | | |  |  |
|  |  | ≥180 days | 59,786 | 6 | | 1.517 | | | 0.955 | | 1.885 | .065 | 24 | 1.478 | 0.999 | | 1.836 | | | | | .052 | | | | |  |  |
|  |  | 180-350 days | 19,868 | 3 | | 2.289 | | | 1.066 | | 2.847 | .001 | 12 | 2.230 | 1.039 | | 2.767 | | | | | .029 | | | | |  |  |
|  |  | 351-667 days | 19,975 | 2 | | 1.134 | | | 0.930 | | 1.406 | .070 | 8 | 1.098 | 0.906 | | 1.361 | | | | | .123 | | | | |  |  |
|  |  | ≥668 days | 19,943 | 1 | | 0.903 | | | 0.538 | | 1.261 | .352 | 4 | 1.110 | 0.911 | | 1.369 | | | | | .201 | | | | |  |  |
| **Hodgkin's disease** | | <180 days | 239,144 | 61 | | Reference | | |  | |  |  | 65 | Reference |  | |  | | | | |  | | | | |  |  |
|  |  | ≥180 days | 59,786 | 18 | | 1.284 | | | 0.991 | | 1.595 | .053 | 33 | 1.243 | 0.967 | | 1.553 | | | | | .074 | | | | |  |  |
|  |  | 180-350 days | 19,868 | 9 | | 2.111 | | | 1.126 | | 2.624 | .001 | 17 | 2.055 | 1.061 | | 2.543 | | | | | .007 | | | | |  |  |
|  |  | 351-667 days | 19,975 | 5 | | 1.002 | | | 0.857 | | 1.293 | .172 | 10 | 1.018 | 0.822 | | 1.222 | | | | | .221 | | | | |  |  |
|  |  | ≥668 days | 19,943 | 4 | | 0.701 | | | 0.576 | | 0.871 | 1.869E-5 | 6 | 0.680 | 0.541 | | 0.832 | | | | | .325 | | | | |  |  |
| **Other malignant neoplasms of lymphoid and histiocytic tissue** | | <180 days | 239,144 | 541 | | Reference | | |  | |  |  | 567 | Reference |  | |  | | | | |  | | | | |  |  |
|  |  | ≥180 days | 59,786 | 68 | | 0.565 | | | 0.465 | | 0.703 | 3.240E-5 | 111 | 0.551 | 0.450 | | 0.687 | | | | | 2.014E-6 | | | | |  |  |
|  |  | 180-350 days | 19,868 | 28 | | 0.732 | | | 0.602 | | 0.909 | 5.171E-4 | 44 | 0.712 | 0.580 | | 0.883 | | | | | 4.025E-6 | | | | |  |  |
|  |  | 351-667 days | 19,975 | 21 | | 0.524 | | | 0.431 | | 0.650 | 6.908E-5 | 39 | 0.508 | 0.416 | | 0.628 | | | | | 4.897E-7 | | | | |  |  |
|  |  | ≥668 days | 19,943 | 19 | | 0.448 | | | 0.366 | | 0.555 | 7.098E-6 | 28 | 0.433 | 0.351 | | 0.546 | | | | | 2.101E-7 | | | | |  |  |
| **Multiple myeloma and immunoproliferative neoplasms** | | <180 days | 239,144 | 369 | | Reference | | |  | |  |  | 387 | Reference |  | | | | | |  | | | |  | |  |  |
|  |  | ≥180 days | 59,786 | 53 | | 0.616 | | | 0.497 | | 0.753 | 6.251E-7 | 65 | 0.606 | 0.498 | 0.748 | | | | | | | 5.522E-7 | | | |  |  |
|  |  | 180-350 days | 19,868 | 19 | | 0.667 | | | 0.548 | | 0.829 | 8.264E-7 | 24 | 0.664 | 0.547 | 0.827 | | | | | | | 7.010E-7 | | | |  |  |
|  |  | 351-667 days | 19,975 | 18 | | 0.603 | | | 0.496 | 0.750 | | 5.707E-8 | 21 | 0.603 | 0.493 | | 0.742 | | | | | | 5.771E-8 | | | |  |  |
|  |  | ≥668 days | 19,943 | 16 | | 0.552 | | | 0.453 | 0.683 | | 2.168E-8 | 20 | 0.550 | 0.455 | | 0.683 | | | | | | 2.014E-8 | | | |  |  |
| **Lymphoid leukemia** | | <180 days | 239,144 | 158 | | Reference | | |  |  | |  | 160 | Reference |  | |  | | | | |  | | | | |  |  |
|  |  | ≥180 days | 59,786 | 25 | | 0.888 | | | 0.608 | 0.998 | | .048 | 38 | 0.891 | 0.609 | | 0.999 | | | | | .049 | | | | |  |  |
|  |  | 180-350 days | 19,868 | 13 | | 1.009 | | | 0.911 | 1.377 | | .172 | 15 | 1.111 | 0.911 | | 1.380 | | | | | .378 | | | | |  |  |
|  |  | 351-667 days | 19,975 | 7 | | 0.549 | | | 0.452 | 0.682 | | 8.752E-4 | 13 | 0.550 | 0.458 | | 0.692 | | | | | 5.214E-5 | | | | |  |  |
|  |  | ≥668 days | 19,943 | 5 | | 0.413 | | | 0.342 | 0.511 | | 3.010E-5 | 10 | 0.416 | 0.344 | | 0.518 | | | | | 3.301E-6 | | | | |  |  |
| **Myeloid leukemia** | | <180 days | 239,144 | 237 | | Reference | | |  |  | |  | 256 | Reference |  | |  | | | | |  | | | | |  |  |
|  |  | ≥180 days | 59,786 | 37 | | 0.707 | | | 0.582 | 0.878 | | 3.892E-5 | 70 | 0.681 | 0.547 | | 0.844 | | | | | 1.098E-4 | | | | |  |  |
|  |  | 180-350 days | 19,868 | 14 | | 0.835 | | | 0.689 | 1.035 | | .067 | 30 | 0.815 | 0.658 | | 0.989 | | | | | .037 | | | | |  |  |
|  |  | 351-667 days | 19,975 | 12 | | 0.735 | | 0.604 | | | 0.911 | 2.307E-5 | 22 | 0.712 | 0.574 | 0.873 | | | | | | 4.801E-5 | | | | |  |  |
|  |  | ≥668 days | 19,943 | 11 | | 0.555 | | 0.457 | | | 0.689 | 9.804E-6 | 18 | 0.538 | 0.430 | 0.664 | | | | | | 2.454E-5 | | | | |  |  |
| **Other specified leukemia** | | <180 days | 239,144 | 17 | | Reference | |  | | |  |  | 27 | Reference |  | |  | | | | | |  | | | |  |  |
|  |  | ≥180 days | 59,786 | 3 | | 0.911 | | 0.742 | | | 1.138 | .310 | 23 | 0.888 | 0.729 | | 1.102 | | | | | | .302 | | | |  |  |
|  |  | 180-350 days | 19,868 | 2 | | 1.372 | | 0.939 | | | 1.705 | .067 | 14 | 1.337 | 0.990 | | 1.662 | | | | | | .084 | | | |  |  |
|  |  | 351-667 days | 19,975 | 1 | | 1.038 | | 0.895 | | | 1.376 | .132 | 6 | 1.324 | 0.872 | | 1.645 | | | | | | .425 | | | |  |  |
|  |  | ≥668 days | 19,943 | 0 | | 0.000 | | - | | | - | .999 | 3 | 0.681 | 0.246 | | 0.854 | | | | | | .719 | | | |  |  |
| **Leukemia of unspecified cell type** | | <180 days | 239,144 | 542 | | Reference |  | | | |  |  | 597 | Reference |  | |  | | | | | | |  | | |  |  |
|  |  | ≥180 days | 59,786 | 90 | | 0.767 | 0.631 | | | | 0.952 | .011 | 163 | 0.767 | 0.632 | | 0.955 | | | | | | | 2.000E-4 | | |  |  |
|  |  | 180-350 days | 19,868 | 32 | | 0.813 | 0.666 | | | | 1.008 | .053 | 66 | 0.811 | 0.667 | | 1.008 | | | | | | | 0.063 | | |  |  |
|  |  | 351-667 days | 19,975 | 30 | | 0.762 | 0.628 | | | | 0.939 | .002 | 57 | 0.763 | 0.627 | | 0.943 | | | | | | | 1.897E-5 | | |  |  |
|  |  | ≥668 days | 19,943 | 28 | | 0.730 | 0.602 | | | | 0.904 | 1.897E-4 | 40 | 0.727 | 0.600 | | 0.909 | | | | | | | 2.587E-6 | | |  |  |
| **Neoplasm of uncertain behavior** | | <180 days | 239,144 | 653 | | Reference | |  | | |  |  | 673 | Reference |  | | |  | | | | | |  | | |  |  |
|  |  | ≥180 days | 59,786 | 101 | | 0.732 | | 0.601 | | | 0.909 | 2.891E-4 | 138 | 0.714 | 0.587 | | | 0.884 | | | | | | 1.606E-6 | | |  |  |
|  |  | 180-350 days | 19,868 | 45 | | 0.969 | | 0.794 | | | 1.213 | .327 | 55 | 0.944 | 0.773 | | 1.157 | | | | | .376 | | | | |  |  |
|  |  | 351-667 days | 19,975 | 32 | | 0.693 | | 0.573 | | | 0.862 | 1.187E-5 | 48 | 0.677 | 0.555 | | 0.841 | | | | | 2.140E-7 | | | | |  |  |
|  |  | ≥668 days | 19,943 | 24 | | 0.532 | | 0.438 | | | 0.666 | 7.267E-5 | 35 | 0.524 | 0.426 | | 0.647 | | | | | 3.297E-8 | | | | |  |  |
| **MDS** | | <180 days | 239,144 | 21 | | Reference | |  | | |  |  | 24 | Reference |  | | |  | | | |  | | | | |  |  |
|  |  | ≥180 days | 59,786 | 5 | | 0.873 | | 0.640 | | | 1.262 | .264 | 19 | 0.875 | 0.706 | | | 1.314 | | | | .352 | | | | |  |  |
|  |  | 180-350 days | 19,868 | 2 | | 1.204 | | 0.805 | | | 1.709 | .479 | 9 | 0.988 | 0.784 | | | 1.359 | | | | .236 | | | | |  |  |
|  |  | 351-667 days | 19,975 | 2 | | 1.168 | | 0.798 | | | 1.648 | .452 | 6 | 0.837 | 0.691 | | | 1.225 | | | | .472 | | | | |  |  |
|  |  | ≥668 days | 19,943 | 1 | | 0.583 | | 0.322 | | | 0.798 | 2.081E-5 | 4 | 0.753 | 0.542 | | | 1.202 | | | | .535 | | | | |  |  |
| **Paraproteinemia** | | <180 days | 239,144 | 203 | | Reference | |  | | |  |  | 222 | Reference |  | | | |  | | |  | | | | |  |  |
|  |  | ≥180 days | 59,786 | 48 | | 1.004 | | 0.878 | | | 1.315 | .168 | 86 | 1.031 | 0.835 | | | | 1.272 | | | .144 | | | | |  |  |
|  |  | 180-350 days | 19,868 | 24 | | 1.596 | | 0.998 | | | 1.982 | .052 | 39 | 1.542 | 0.970 | | | | 1.919 | | | .251 | | | | |  |  |
|  |  | 351-667 days | 19,975 | 14 | | 0.973 | | 0.779 | | | 1.174 | .247 | 28 | 0.924 | 0.753 | | | | 1.139 | | | .398 | | | | |  |  |
|  |  | ≥668 days | 19,943 | 10 | | 0.636 | | 0.523 | | | 0.791 | 5.232E-5 | 19 | 0.616 | 0.505 | | | | 0.762 | | | 5.201E-7 | | | | |  |  |
| **Other polycythemia** | | <180 days | 239,144 | 1,722 | | Reference | |  | | |  |  | 2,017 | Reference |  | | | | |  | | | |  | | | | |
|  |  | ≥180 days | 59,786 | 303 | | 0.802 | | 0.658 | | | 0.901 | 1.976E-8 | 598 | 0.770 | 0.633 | | | | | 0.954 | | | | 2.067E-4 | | | | |
|  |  | 180-350 days | 19,868 | 103 | | 0.816 | | 0.672 | | | 0.937 | 3.201E-8 | 246 | 0.783 | 0.644 | | | | | 0.976 | | | | 3.454E-4 | | | | |
|  |  | 351-667 days | 19,975 | 101 | | 0.797 | | 0.653 | | | 0.865 | 9.478E-9 | 198 | 0.765 | 0.628 | | | | | 0.949 | | | | 5.009E-5 | | | | |
|  |  | ≥668 days | 19,943 | 99 | | 0.789 | | 0.648 | | | 0.835 | 7.067E-9 | 154 | 0.758 | 0.619 | | | | | 0.923 | | | | 4.097E-6 | | | | |
| Abbreviations: CI, confidence interval; MDS, myelodysplastic syndrome.  The subgroup of monocytic leukemia is not listed due to the lack of events in the first-event model.  † Competing variable was all-cause mortality.  * Proportional-hazards assumption test was checked based on Schoenfeld residuals. Global test: *P* = 0.764 (first-event model), *P* = 0.725 (multiple-event model, Lymphosarcoma and reticulosarcoma), *P* = 0.684 (multiple-event model, Hodgkin's disease), P = 0.731 (multiple-event model, Other malignant neoplasms of lymphoid and histiocytic tissue), *P* = 0.583 (multiple-event model, Multiple myeloma and immunoproliferative neoplasms), P = 0.811 (multiple-event model, Lymphoid leukemia), *P* = 0.472 (multiple-event model, Myeloid leukemia), P = 0.530 (multiple-event model, Monocytic leukemia), P = 0.679 (multiple-event model, Other specified leukemia), *P* = 0.354 (multiple-event model, Leukemia of unspecified cell type), *P* = 0.671 (multiple-event model, Neoplasm of uncertain behavior), *P* = 0.863 (multiple-event model, MDS), *P* = 0.575 (multiple-event model, Paraproteinemia), *P* = 0.698 (multiple-event model, Other polycythemia)  § Adjusted sHR = adjusted subdistribution hazard ratio; All variables controlled by the model (§) include demographics (sex, age, insured premium, location, urbanization level, and level of hospital), comorbidities (congestive heart failure, pulmonary embolism, gastrointestinal hemorrhage, cerebral thrombosis, ischemic heart disease, vascular insufficiency of intestine, obesity, malignant neoplasm of kidney/renal pelvis, acute glomerulonephritis/nephrotic syndrome, proteinuria, gestational hypertension, and asthma), other variables (normal pregnancy and Charlson Comorbidity Index_Revised), and medications (aspirin, celecoxib, itraconazole, mebendazole, leflunomide, thalidomide, valproate, metformin, auranofin, statins [nystatin, lovastatin, pravastatin, simvastatin, atorvastatin, pitavastatin, rosuvastatin, cilastatin], bisphosphonates [alendronate and risedronate], bromocriptine, chlorprothixene, clotrimazole, quinacrine, ivermectin, verteporfin, clarithromycin, hydroxychloroquine, tofacitinib, gefitinib, curcumin, chlorhexidine, and axitinib). | | | | | | | | | | | | | | | | | | | | | | | | | | | |  |

| **Table D. Multivariable Risk Regression Analysis of Hematologic Neoplasm Development in Patients without/ with Hypertension in Competing Risk Model*** | | | | | | | | |
| --- | --- | --- | --- | --- | --- | --- | --- | --- |
| **Cohorts** | **No Competing Risk Model** | | | | **Fine and Gray's Competing Risk Model**† | | | |
|  | **Adjusted HR ‡** | **95% CI** | **95% CI** | ***P*** | **Adjusted sHR** § | **95% CI** | **95% CI** | ***P*** |
| Patient without Hypertension | Reference |  |  |  | Reference |  |  |  |
| Patients with Hypertension | 1.512 | 1.415 | 1.672 | 5.786E-5 | 1.483 | 1.397 | 1.654 | 3.012E-5 |
| Hydralazine < 180 days | 1.985 | 1.857 | 2.038 | 7.301E-6 | 1.924 | 1.830 | 2.001 | 9.486E-5 |
| Hydralazine ≥180 days | 1.153 | 1.080 | 1.216 | 0.010 | 1.119 | 1.064 | 1.187 | 0.018 |
| Abbreviations: HR = hazard ratio; CI = confidence interval  Patients without Hypertension: Patients with Hypertension = 4:1, 4-fold propensity score matching by age, sex, and season of index date  * Proportional-hazards assumption test was checked based on Schoenfeld residuals. Global test: *P* =0.714 (without competing), *P* =0.653 (with competing).  † Competing variable was all-cause mortality.  ‡ Adjusted HR = adjusted hazard ratio; § Adjusted sHR = adjusted subdistribution hazard ratio; All variables controlled by the models (‡ and §) include demographics (sex, age, insured premium, location, urbanization level, and level of hospital), comorbidities (congestive heart failure, pulmonary embolism, gastrointestinal hemorrhage, cerebral thrombosis, ischemic heart disease, vascular insufficiency of intestine, obesity, malignant neoplasm of kidney/renal pelvis, acute glomerulonephritis/nephrotic syndrome, proteinuria, gestational hypertension, and asthma), other variables (normal pregnancy and Charlson Comorbidity Index_Revised), and medications (aspirin, celecoxib, itraconazole, mebendazole, leflunomide, thalidomide, valproate, metformin, auranofin, statins [nystatin, lovastatin, pravastatin, simvastatin, atorvastatin, pitavastatin, rosuvastatin, cilastatin], bisphosphonates [alendronate and risedronate], bromocriptine, chlorprothixene, clotrimazole, quinacrine, ivermectin, verteporfin, clarithromycin, hydroxychloroquine, tofacitinib, gefitinib, curcumin, chlorhexidine, and axitinib). | | | | | | | | |

| **Table E. Adjusted Hazard Ratio for Remaining Hematologic Neoplasm Subgroups, Stratified by Prescription Duration of Hydralazine** | | | | | | | | | |
| --- | --- | --- | --- | --- | --- | --- | --- | --- | --- |
| **Subgroups of Hematologic Neoplasms** | **Prescription Duration of Hydralazine** | **Population** | **Events** | **PYs** | **Rate (per 10^5^ PYs)** | **Adjusted HR**‡ | **95% CI** | | ***P*** |
| Lymphosarcoma and reticulosarcoma | < 180 days | 239,144 | 20 | 2,834,197.06 | 0.71 | Reference |  |  |  |
|  | ≥ 180 days | 59,786 | 6 | 716,983.56 | 0.84 | 1.498 | 0.982 | 1.861 | .067 |
|  | 180-350 days | 19,868 | 3 | 238,267.67 | 1.26 | 2.257 | 1.052 | 2.804 | .004 |
|  | 351-667 days | 19,975 | 2 | 239,805.11 | 0.83 | 1.118 | 0.918 | 1.388 | .078 |
|  | ≥668 days | 19,943 | 1 | 238,910.78 | 0.42 | 0.892 | 0.531 | 1.245 | .452 |
| Hodgkin's disease | < 180 days | 239,144 | 61 | 2,834,197.06 | 2.15 | Reference |  |  |  |
|  | ≥180 days | 59,786 | 18 | 716,983.56 | 2.51 | 1.268 | 0.978 | 1.575 | .057 |
|  | 180-350 days | 19,868 | 9 | 238,267.67 | 3.78 | 2.084 | 1.112 | 2.588 | .001 |
|  | 351-667 days | 19,975 | 5 | 239,805.11 | 2.09 | 0.989 | 0.847 | 1.281 | .172 |
|  | ≥668 days | 19,943 | 4 | 238,910.78 | 1.67 | 0.692 | 0.569 | 0.860 | < .001 |
| Lymphoid leukemia | < 180 days | 239,144 | 158 | 2,834,197.06 | 5.57 | Reference |  |  |  |
|  | ≥180 days | 59,786 | 25 | 716,983.56 | 3.49 | 0.876 | 0.598 | 0.986 | .028 |
|  | 180-350 days | 19,868 | 13 | 238,267.67 | 5.46 | 0.995 | 0.899 | 1.359 | .189 |
|  | 351-667 days | 19,975 | 7 | 239,805.11 | 2.92 | 0.542 | 0.445 | 0.673 | < .001 |
|  | ≥668 days | 19,943 | 5 | 238,910.78 | 2.09 | 0.409 | 0.336 | 0.508 | < .001 |
| Myeloid leukemia | < 180 days | 239,144 | 237 | 2,834,197.06 | 8.36 | Reference |  |  |  |
|  | ≥180 days | 59,786 | 37 | 716,983.56 | 5.16 | 0.698 | 0.574 | 0.868 | < .001 |
|  | 180-350 days | 19,868 | 14 | 238,267.67 | 5.88 | 0.824 | 0.677 | 1.023 | .073 |
|  | 351-667 days | 19,975 | 12 | 239,805.11 | 5.00 | 0.725 | 0.596 | 0.900 | < .001 |
|  | ≥668 days | 19,943 | 11 | 238,910.78 | 4.60 | 0.547 | 0.450 | 0.680 | < .001 |
| Other specified leukemia | < 180 days | 239,144 | 17 | 2,834,197.06 | 0.60 | Reference |  |  |  |
|  | ≥180 days | 59,786 | 3 | 716,983.56 | 0.42 | 0.899 | 0.739 | 1.117 | .316 |
|  | 180-350 days | 19,868 | 2 | 238,267.67 | 0.84 | 1.354 | 0.928 | 1.682 | .078 |
|  | 351-667 days | 19,975 | 1 | 239,805.11 | 0.42 | 1.025 | 0.884 | 1.356 | .184 |
|  | ≥668 days | 19,943 | 0 | 238,910.78 | 0.00 | 0.000 | - | - | .999 |
| Leukemia of unspecified cell type | < 180 days | 239,144 | 542 | 2,834,197.06 | 19.12 | Reference |  |  |  |
|  | ≥180 days | 59,786 | 90 | 716,983.56 | 12.55 | 0.758 | 0.623 | 0.942 | .008 |
|  | 180-350 days | 19,868 | 32 | 238,267.67 | 13.43 | 0.802 | 0.659 | 0.995 | .046 |
|  | 351-667 days | 19,975 | 30 | 239,805.11 | 12.51 | 0.754 | 0.619 | 0.936 | .002 |
|  | ≥668 days | 19,943 | 28 | 238,910.78 | 11.72 | 0.719 | 0.591 | 0.893 | < .001 |
| Neoplasm of uncertain behavior | < 180 days | 239,144 | 653 | 2,834,197.06 | 23.04 | Reference |  |  |  |
|  | ≥180 days | 59,786 | 101 | 716,983.56 | 14.09 | 0.722 | 0.593 | 0.897 | < .001 |
|  | 180-350 days | 19,868 | 45 | 238,267.67 | 18.89 | 0.956 | 0.785 | 1.187 | .332 |
|  | 351-667 days | 19,975 | 32 | 239,805.11 | 13.34 | 0.685 | 0.564 | 0.851 | < .001 |
|  | ≥668 days | 19,943 | 24 | 238,910.78 | 10.05 | 0.526 | 0.432 | 0.653 | < .001 |
| MDS | < 180 days | 239,144 | 21 | 2,834,197.06 | 0.74 | Reference |  |  |  |
|  | ≥180 days | 59,786 | 5 | 716,983.56 | 0.70 | 0.861 | 0.632 | 1.246 | .279 |
|  | 180-350 days | 19,868 | 2 | 238,267.67 | 0.84 | 1.189 | 0.794 | 1.686 | .488 |
|  | 351-667 days | 19,975 | 2 | 239,805.11 | 0.83 | 1.154 | 0.789 | 1.625 | .473 |
|  | ≥668 days | 19,943 | 1 | 238,910.78 | 0.42 | 0.576 | 0.320 | 0.786 | < .001 |
| Paraproteinemia | < 180 days | 239,144 | 203 | 2,834,197.06 | 7.16 | Reference |  |  |  |
|  | ≥180 days | 59,786 | 48 | 716,983.56 | 6.69 | 0.989 | 0.859 | 1.298 | .172 |
|  | 180-350 days | 19,868 | 24 | 238,267.67 | 10.07 | 1.575 | 0.992 | 1.956 | .058 |
|  | 351-667 days | 19,975 | 14 | 239,805.11 | 5.84 | 0.935 | 0.769 | 1.162 | .298 |
|  | ≥668 days | 19,943 | 10 | 238,910.78 | 4.19 | 0.628 | 0.516 | 0.780 | < .001 |
| Abbreviations: PYs = person-years; CI = confidence interval; MDS = myelodysplastic syndrome.  The subgroup of monocytic leukemia is not listed due to the lack of events.  ‡ Adjusted HR = adjusted hazard ratio; All variables controlled by the model (‡) include demographics (sex, age, insured premium, location, urbanization level, and level of hospital), comorbidities (congestive heart failure, pulmonary embolism, gastrointestinal hemorrhage, cerebral thrombosis, ischemic heart disease, vascular insufficiency of intestine, obesity, malignant neoplasm of kidney/renal pelvis, acute glomerulonephritis/nephrotic syndrome, proteinuria, gestational hypertension, and asthma), other variables (normal pregnancy and Charlson Comorbidity Index_Revised), and medications (aspirin, celecoxib, itraconazole, mebendazole, leflunomide, thalidomide, valproate, metformin, auranofin, statins [nystatin, lovastatin, pravastatin, simvastatin, atorvastatin, pitavastatin, rosuvastatin, cilastatin], bisphosphonates [alendronate and risedronate], bromocriptine, chlorprothixene, clotrimazole, quinacrine, ivermectin, verteporfin, clarithromycin, hydroxychloroquine, tofacitinib, gefitinib, curcumin, chlorhexidine, and axitinib). | | | | | | | | | |

| \| **Table F. Leave-One-Out Analysis for Comparison of Adjusted Hazard Ratio of Hematologic Neoplasms According to Subgroup Stratified by Prescription Duration of Hydralazine in First-Event and Multiple-Event Models in a Competing Risk Model** *† \| \| \| \| \| \| \| \| \| \| \| \| --- \| --- \| --- \| --- \| --- \| --- \| --- \| --- \| --- \| --- \| --- \| \| **Subgroups of Hematologic Neoplasms** \| **Prescription Duration of Hydralazine** \| **First-Event Model** \| \| \| \| **Multiple-Event Model** \| \| \| \| \| **Adjusted sHR**§ \| **95% CI** \| **95% CI** \| ***P*** \| **Adjusted sHR**§ \| **95% CI** \| **95% CI** \| ***P*** \| \| **Overall (Hematologic neoplasms)** \| <180 days \| Reference \|  \|  \|  \| - \| - \| - \| - \| \| ≥180 days \| 0.751 \| 0.636 \| 0.894 \| 7.014E-5 \| - \| - \| - \| - \| \| 180-350 days \| 0.871 \| 0.724 \| 1.094 \| .213 \| - \| - \| - \| - \| \| 351-667 days \| 0.717 \| 0.583 \| 0.901 \| .001 \| - \| - \| - \| - \| \| ≥668 days \| 0.636 \| 0.517 \| 0.800 \| 8.241E-6 \| - \| - \| - \| - \| \| **Lymphosarcoma and reticulosarcoma** \| <180 days \| Reference \|  \|  \|  \| Reference \|  \|  \|  \| \| ≥180 days \| 1.476 \| 0.957 \| 1.854 \| .182 \| 1.440 \| 0.973 \| 1.788 \| .297 \| \| 180-350 days \| 2.224 \| 1.025 \| 2.794 \| .037 \| 2.172 \| 1.012 \| 2.695 \| .036 \| \| 351-667 days \| 1.101 \| 0.894 \| 1.383 \| .114 \| 1.070 \| 0.882 \| 1.326 \| .181 \| \| ≥668 days \| 0.879 \| 0.517 \| 1.240 \| .536 \| 1.082 \| 0.887 \| 1.333 \| .305 \| \| **Hodgkin's disease** \| <180 days \| Reference \|  \|  \|  \| Reference \|  \|  \|  \| \| ≥180 days \| 1.249 \| 0.953 \| 1.569 \| .062 \| 1.211 \| 0.942 \| 1.513 \| .097 \| \| 180-350 days \| 2.053 \| 1.083 \| 2.578 \| .009 \| 2.002 \| 1.033 \| 2.477 \| .025 \| \| 351-667 days \| 0.974 \| 0.825 \| 1.276 \| .164 \| 0.992 \| 0.800 \| 1.190 \| .279 \| \| ≥668 days \| 0.682 \| 0.554 \| 0.857 \| 1.870E-5 \| 0.662 \| 0.527 \| 0.810 \| .384 \| \| **Other malignant neoplasms of lymphoid and histiocytic tissue** \| <180 days \| Reference \|  \|  \|  \| Reference \|  \|  \|  \| \| ≥180 days \| 0.550 \| 0.447 \| 0.691 \| 3.795E-5 \| 0.537 \| 0.438 \| 0.669 \| 3.892E-6 \| \| 180-350 days \| 0.711 \| 0.578 \| 0.893 \| 5.598E-4 \| 0.694 \| 0.565 \| 0.861 \| 4.895E-6 \| \| 351-667 days \| 0.508 \| 0.413 \| 0.639 \| 7.135E-5 \| 0.494 \| 0.406 \| 0.612 \| 5.201E-7 \| \| ≥668 days \| 0.433 \| 0.352 \| 0.543 \| 8.802E-6 \| 0.421 \| 0.342 \| 0.532 \| 3.010E-7 \| \| **Multiple myeloma and immunoproliferative neoplasms** \| <180 days \| Reference \|  \|  \|  \| Reference \|  \|  \|  \| \| ≥180 days \| 0.589 \| 0.479 \| 0.740 \| 8.201E-7 \| 0.590 \| 0.486 \| 0.728 \| 5.895E-7 \| \| 180-350 days \| 0.646 \| 0.524 \| 0.811 \| 1.256E-5 \| 0.646 \| 0.533 \| 0.805 \| 7.502E-7 \| \| 351-667 days \| 0.586 \| 0.476 \| 0.736 \| 6.883E-8 \| 0.587 \| 0.481 \| 0.722 \| 6.890E-8 \| \| ≥668 days \| 0.536 \| 0.435 \| 0.673 \| 3.798E-8 \| 0.536 \| 0.443 \| 0.665 \| 3.504E-8 \| \| **Lymphoid leukemia** \| <180 days \| Reference \|  \|  \|  \| Reference \|  \|  \|  \| \| ≥180 days \| 0.863 \| 0.583 \| 0.982 \| .041 \| 0.868 \| 0.593 \| 0.976 \| .035 \| \| 180-350 days \| 0.980 \| 0.876 \| 1.354 \| .231 \| 1.083 \| 0.887 \| 1.344 \| .484 \| \| 351-667 days \| 0.534 \| 0.433 \| 0.671 \| 8.201E-4 \| 0.536 \| 0.446 \| 0.674 \| 7.501E-5 \| \| ≥668 days \| 0.403 \| 0.327 \| 0.506 \| 7.012E-5 \| 0.406 \| 0.336 \| 0.504 \| 5.050E-6 \| \| **Myeloid leukemia** \| <180 days \| Reference \|  \|  \|  \| Reference \|  \|  \|  \| \| ≥180 days \| 0.688 \| 0.559 \| 0.865 \| 4.972E-5 \| 0.663 \| 0.533 \| 0.822 \| 2.974E-4 \| \| 180-350 days \| 0.812 \| 0.659 \| 1.019 \| .085 \| 0.794 \| 0.641 \| 0.974 \| .025 \| \| 351-667 days \| 0.714 \| 0.581 \| 0.897 \| 4.013E-5 \| 0.694 \| 0.560 \| 0.851 \| 5.971E-5 \| \| ≥668 days \| 0.539 \| 0.438 \| 0.677 \| 1.255E-5 \| 0.524 \| 0.418 \| 0.646 \| 3.287E-5 \| \| **Monocytic leukemia** \| <180 days \| Reference \|  \|  \|  \| Reference \|  \|  \|  \| \| ≥180 days \| - \| - \| - \| - \| 0.885 \| 0.569 \| 1.949 \| .489 \| \| 180-350 days \| - \| - \| - \| - \| 0.933 \| 0.727 \| 1.985 \| .234 \| \| 351-667 days \| - \| - \| - \| - \| 0.677 \| 0.417 \| 1.368 \| .553 \| \| ≥668 days \| - \| - \| - \| - \| 0.425 \| 0.132 \| 2.859 \| .895 \| \| **Other specified leukemia** \| <180 days \| Reference \|  \|  \|  \| Reference \|  \|  \|  \| \| ≥180 days \| 0.886 \| 0.720 \| 1.113 \| .421 \| 0.865 \| 0.711 \| 1.074 \| .345 \| \| 180-350 days \| 1.334 \| 0.904 \| 1.676 \| .186 \| 1.303 \| 0.964 \| 1.619 \| .125 \| \| 351-667 days \| 1.010 \| 0.861 \| 1.351 \| .202 \| 1.290 \| 0.850 \| 1.603 \| .446 \| \| ≥668 days \| 0.000 \| - \| - \| .999 \| 0.663 \| 0.240 \| 0.832 \| .784 \| \| **Leukemia of unspecified cell type** \| <180 days \| Reference \|  \|  \|  \| Reference \|  \|  \|  \| \| ≥180 days \| 0.747 \| 0.607 \| 0.939 \| .020 \| 0.747 \| 0.616 \| 0.931 \| 2.564E-4 \| \| 180-350 days \| 0.790 \| 0.642 \| 0.991 \| .045 \| 0.790 \| 0.649 \| 1.004 \| .068 \| \| 351-667 days \| 0.743 \| 0.603 \| 0.933 \| .016 \| 0.743 \| 0.611 \| 0.919 \| 2.303E-5 \| \| ≥668 days \| 0.708 \| 0.576 \| 0.890 \| 1.873E-4 \| 0.709 \| 0.584 \| 0.885 \| 4.560E-6 \| \| **Neoplasm of uncertain behavior** \| <180 days \| Reference \|  \|  \|  \| Reference \|  \|  \|  \| \| ≥180 days \| 0.711 \| 0.578 \| 0.894 \| 4.532E-4 \| 0.696 \| 0.571 \| 0.862 \| 3.897E-6 \| \| 180-350 days \| 0.942 \| 0.765 \| 1.183 \| .423 \| 0.920 \| 0.753 \| 1.127 \| .386 \| \| 351-667 days \| 0.675 \| 0.549 \| 0.848 \| 2.597E-5 \| 0.659 \| 0.541 \| 0.819 \| 4.894E-7 \| \| ≥668 days \| 0.518 \| 0.421 \| 0.651 \| 8.707E-5 \| 0.510 \| 0.415 \| 0.631 \| 5.100E-8 \| \| **MDS** \| <180 days \| Reference \|  \|  \|  \| Reference \|  \|  \|  \| \| ≥180 days \| 0.848 \| 0.616 \| 1.241 \| .301 \| 0.853 \| 0.688 \| 1.280 \| .379 \| \| 180-350 days \| 1.171 \| 0.773 \| 1.680 \| .492 \| 0.962 \| 0.764 \| 1.324 \| .275 \| \| 351-667 days \| 1.137 \| 0.769 \| 1.619 \| .465 \| 0.815 \| 0.673 \| 1.193 \| .484 \| \| ≥668 days \| 0.567 \| 0.312 \| 0.783 \| 6.121E-5 \| 0.733 \| 0.528 \| 1.171 \| .552 \| \| **Paraproteinemia** \| <180 days \| Reference \|  \|  \|  \| Reference \|  \|  \|  \| \| ≥180 days \| 0.974 \| 0.837 \| 1.293 \| .166 \| 1.005 \| 0.813 \| 1.239 \| .186 \| \| 180-350 days \| 1.552 \| 0.966 \| 1.949 \| .064 \| 1.502 \| 0.945 \| 1.869 \| .267 \| \| 351-667 days \| 0.921 \| 0.749 \| 1.158 \| .301 \| 0.900 \| 0.733 \| 1.109 \| .422 \| \| ≥668 days \| 0.619 \| 0.503 \| 0.777 \| 5.673E-5 \| 0.600 \| 0.492 \| 0.742 \| 6.124E-7 \| \| **Familial polycythemia** \| <180 days \| Reference \|  \|  \|  \| Reference \|  \|  \|  \| \| ≥180 days \| 0.892 \| 0.432 \| 1.210 \| .625 \| 0.872 \| 0.452 \| 1.186 \| .421 \| \| 180-350 days \| 0.733 \| 0.409 \| 1.152 \| .603 \| 0.633 \| 0.341 \| 1.035 \| .386 \| \| 351-667 days \| - \| - \| - \| - \| 0.891 \| 0.479 \| 1.203 \| .479 \| \| ≥668 days \| 0.975 \| 0.588 \| 1.397 \| .666 \| 0.972 \| 0.502 \| 1.288 \| .511 \| \| Abbreviations: PYs = Person-years; CI = confidence interval  † Competing variable was all-cause mortality.  * Proportional-hazards assumption test was checked based on Schoenfeld residuals. Global test: *P* = 0.530 (first-event model), *P* = 0.725 (multiple-event model, Lymphosarcoma and reticulosarcoma), *P* = 0.684 (multiple-event model, Hodgkin's disease), *P* = 0.731 (multiple-event model, Other malignant neoplasms of lymphoid and histiocytic tissue), *P* = 0.583 (multiple-event model, Multiple myeloma and immunoproliferative neoplasms), *P* = 0.811 (multiple-event model, Lymphoid leukemia), *P* = 0.472 (multiple-event model, Myeloid leukemia), *P* = 0.530 (multiple-event model, Monocytic leukemia), *P* = 0.679 (multiple-event model, Other specified leukemia), *P* = 0.354 (multiple-event model, Leukemia of unspecified cell type), *P* = 0.671 (multiple-event model, Neoplasm of uncertain behavior), *P* = 0.863 (multiple-event model, MDS), *P* = 0.575 (multiple-event model, Paraproteinemia), *P* = 0.606 (multiple-event model, Other polycythemia)  § Adjusted sHR = adjusted subdistribution hazard ratio; All variables controlled by the model (§) include demographics (sex, age, insured premium, location, urbanization level, and level of hospital), comorbidities (congestive heart failure, pulmonary embolism, gastrointestinal hemorrhage, cerebral thrombosis, ischemic heart disease, vascular insufficiency of intestine, obesity, malignant neoplasm of kidney/renal pelvis, acute glomerulonephritis/nephrotic syndrome, proteinuria, gestational hypertension, and asthma), other variables (normal pregnancy and Charlson Comorbidity Index_Revised), and medications (aspirin, celecoxib, itraconazole, mebendazole, leflunomide, thalidomide, valproate, metformin, auranofin, statins [nystatin, lovastatin, pravastatin, simvastatin, atorvastatin, pitavastatin, rosuvastatin, cilastatin], bisphosphonates [alendronate and risedronate], bromocriptine, chlorprothixene, clotrimazole, quinacrine, ivermectin, verteporfin, clarithromycin, hydroxychloroquine, tofacitinib, gefitinib, curcumin, chlorhexidine, and axitinib). \| \| \| \| \| \| \| \| \| \| \| |  |
| --- | --- | --- | --- | --- | --- | --- | --- | --- | --- | --- | --- | --- | --- | --- | --- | --- | --- | --- | --- | --- | --- | --- | --- | --- | --- | --- | --- | --- | --- | --- | --- | --- | --- | --- | --- | --- | --- | --- | --- | --- | --- | --- | --- | --- | --- | --- | --- | --- | --- | --- | --- | --- | --- | --- | --- | --- | --- | --- | --- | --- | --- | --- | --- | --- | --- | --- | --- | --- | --- | --- | --- | --- | --- | --- | --- | --- | --- | --- | --- | --- | --- | --- | --- | --- | --- | --- | --- | --- | --- | --- | --- | --- | --- | --- | --- | --- | --- | --- | --- | --- | --- | --- | --- | --- | --- | --- | --- | --- | --- | --- | --- | --- | --- | --- | --- | --- | --- | --- | --- | --- | --- | --- | --- | --- | --- | --- | --- | --- | --- | --- | --- | --- | --- | --- | --- | --- | --- | --- | --- | --- | --- | --- | --- | --- | --- | --- | --- | --- | --- | --- | --- | --- | --- | --- | --- | --- | --- | --- | --- | --- | --- | --- | --- | --- | --- | --- | --- | --- | --- | --- | --- | --- | --- | --- | --- | --- | --- | --- | --- | --- | --- | --- | --- | --- | --- | --- | --- | --- | --- | --- | --- | --- | --- | --- | --- | --- | --- | --- | --- | --- | --- | --- | --- | --- | --- | --- | --- | --- | --- | --- | --- | --- | --- | --- | --- | --- | --- | --- | --- | --- | --- | --- | --- | --- | --- | --- | --- | --- | --- | --- | --- | --- | --- | --- | --- | --- | --- | --- | --- | --- | --- | --- | --- | --- | --- | --- | --- | --- | --- | --- | --- | --- | --- | --- | --- | --- | --- | --- | --- | --- | --- | --- | --- | --- | --- | --- | --- | --- | --- | --- | --- | --- | --- | --- | --- | --- | --- | --- | --- | --- | --- | --- | --- | --- | --- | --- | --- | --- | --- | --- | --- | --- | --- | --- | --- | --- | --- | --- | --- | --- | --- | --- | --- | --- | --- | --- | --- | --- | --- | --- | --- | --- | --- | --- | --- | --- | --- | --- | --- | --- | --- | --- | --- | --- | --- | --- | --- | --- | --- | --- | --- | --- | --- | --- | --- | --- | --- | --- | --- | --- | --- | --- | --- | --- | --- | --- | --- | --- | --- | --- | --- | --- | --- | --- | --- | --- | --- | --- | --- | --- | --- | --- | --- | --- | --- | --- | --- | --- | --- | --- | --- | --- | --- | --- | --- | --- | --- | --- | --- | --- | --- | --- | --- | --- | --- | --- | --- | --- | --- | --- | --- | --- | --- | --- | --- | --- | --- | --- | --- | --- | --- | --- | --- | --- | --- | --- | --- | --- | --- | --- | --- | --- | --- | --- | --- | --- | --- | --- | --- | --- | --- | --- | --- | --- | --- | --- | --- | --- | --- | --- | --- | --- | --- | --- | --- | --- | --- | --- | --- | --- | --- | --- | --- | --- | --- | --- | --- | --- | --- | --- | --- | --- | --- | --- | --- | --- | --- | --- | --- | --- | --- | --- | --- | --- | --- | --- | --- | --- | --- | --- | --- | --- | --- | --- | --- | --- | --- | --- | --- | --- | --- | --- | --- | --- | --- | --- | --- | --- | --- | --- | --- | --- | --- | --- | --- | --- | --- | --- | --- | --- | --- | --- | --- | --- | --- | --- | --- | --- | --- | --- | --- | --- | --- | --- | --- | --- | --- | --- | --- | --- | --- | --- | --- | --- | --- | --- | --- | --- | --- | --- | --- | --- | --- | --- | --- | --- | --- | --- | --- | --- | --- | --- | --- | --- | --- | --- | --- | --- | --- | --- | --- | --- | --- | --- | --- | --- | --- | --- | --- | --- | --- | --- | --- | --- | --- | --- | --- | --- | --- | --- | --- | --- | --- | --- | --- | --- | --- | --- | --- | --- | --- | --- | --- | --- | --- | --- | --- | --- | --- | --- | --- | --- | --- | --- | --- | --- | --- | --- | --- | --- | --- | --- | --- | --- | --- | --- | --- | --- | --- | --- | --- | --- | --- | --- | --- | --- | --- | --- | --- | --- | --- | --- | --- | --- | --- | --- | --- | --- | --- | --- | --- | --- | --- | --- | --- | --- | --- | --- | --- | --- | --- | --- | --- | --- | --- | --- | --- | --- | --- | --- | --- | --- | --- | --- | --- | --- | --- | --- | --- | --- | --- | --- | --- | --- | --- | --- | --- | --- | --- | --- | --- | --- | --- | --- | --- | --- | --- | --- | --- | --- | --- | --- | --- | --- | --- |
| \| **Table G. Tracking Years in Patients with Hypertension by Prescription Duration of Hydralazine** \| \| \| \| \| \| \| --- \| --- \| --- \| --- \| --- \| --- \| \| **Hydralazine** \| **Min** \| **Median** \| **Max** \| **Mean ± SD** \| ***P*** \| \| Overall \| 0.01 \| 9.18 \| 15.58 \| 10.08 ± 8.46 \| .722 \| \| <180 days \| 0.01 \| 9.09 \| 15.29 \| 10.03 ± 8.31 \|  \| \| ≥180 days \| 0.01 \| 9.46 \| 15.58 \| 10.14 ± 8.65 \|  \| \| ***P*: t-test** \| \| \| \| \| \|   Abbreviations: SD, standard deviation.   \| **Table H. Tracking Years from Initiating Hydralazine Prescription to** **Having Hematologic Neoplasms in Patients with Hypertension** \| \| \| \| \| \| \| --- \| --- \| --- \| --- \| --- \| --- \| \| **Hydralazine** \| **Min** \| **Median** \| **Max** \| **Mean ± SD** \| ***P*** \| \| Overall \| 0.58 \| 7.26 \| 15.58 \| 8.10 ± 7.91 \| 9.782E-5 \| \| <180 days \| 0.94 \| 7.05 \| 15.29 \| 7.96 ± 7.82 \|  \| \| ≥180 days \| 0.58 \| 7.33 \| 15.58 \| 8.18 ± 7.99 \|  \| \| ***P*: Mann-Whitney U test** \| \| \| \| \| \| \| Abbreviations: SD, standard deviation. \| \| \| \| \| \|   **Table I. Endpoint Characteristics of Patients with Hypertension by Prescription Duration of Hydralazine, 2000–2015**   \| **Hydralazine** \| **Overall** \| \|  \| **<180 days** \| \|  \| **≥180 days** \| \| ***P*** \| \| --- \| --- \| --- \| --- \| --- \| --- \| --- \| --- \| --- \| --- \| \| **Variables** \| **n** \| **%** \|  \| **n** \| **%** \|  \| **n** \| **%** \| \| **Total** \| 298,930 \|  \|  \| 239,144 \|  \|  \| 59,786 \|  \|  \| \| **Hematologic neoplasm** \| 5,301 \| 1.77 \|  \| 4,544 \| 1.90 \|  \| 757 \| 1.27 \| 1.383E-5 \| \| Lymphosarcoma and reticulosarcoma \| 26 \| 0.01 \|  \| 20 \| 0.01 \|  \| 6 \| 0.01 \| .989 \| \| Hodgkin's disease \| 79 \| 0.03 \|  \| 61 \| 0.03 \|  \| 18 \| 0.03 \| .972 \| \| Other malignant neoplasms of lymphoid and histiocytic tissue \| 609 \| 0.20 \|  \| 541 \| 0.23 \|  \| 68 \| 0.11 \| 3.762E-10 \| \| Multiple myeloma and immunoproliferative neoplasms \| 422 \| 0.14 \|  \| 369 \| 0.15 \|  \| 53 \| 0.09 \| 4.003E-9 \| \| Lymphoid leukemia \| 183 \| 0.06 \|  \| 158 \| 0.07 \|  \| 25 \| 0.04 \| .075 \| \| Myeloid leukemia \| 274 \| 0.09 \|  \| 237 \| 0.10 \|  \| 37 \| 0.06 \| .027 \| \| Monocytic leukemia \| 0 \| 0.00 \|  \| 0 \| 0.00 \|  \| 0 \| 0.00 \| - \| \| Other specified leukemia \| 20 \| 0.01 \|  \| 17 \| 0.01 \|  \| 3 \| 0.01 \| .923 \| \| Leukemia of unspecified cell type \| 632 \| 0.21 \|  \| 542 \| 0.23 \|  \| 90 \| 0.15 \| 2.951E-6 \| \| Neoplasm of uncertain behavior \| 754 \| 0.25 \|  \| 653 \| 0.27 \|  \| 101 \| 0.17 \| 3.307E-8 \| \| MDS \| 26 \| 0.01 \|  \| 21 \| 0.01 \|  \| 5 \| 0.01 \| .864 \| \| Paraproteinemia \| 251 \| 0.08 \|  \| 203 \| 0.08 \|  \| 48 \| 0.08 \| .986 \| \| Other polycythemia \| 2,025 \| 0.68 \|  \| 1,722 \| 0.72 \|  \| 303 \| 0.51 \| 8.064E-10 \| \| Polycythemia, secondary \| 2,005 \| 0.67 \|  \| 1,705 \| 0.71 \|  \| 300 \| 0.50 \| 2.013E-9 \| \| Familial polycythemia \| 20 \| 0.01 \|  \| 17 \| 0.01 \|  \| 3 \| 0.01 \| .782 \| \| **Sex** \|  \|  \|  \|  \|  \|  \|  \|  \| .999 \| \| Male \| 156,000 \| 52.19 \|  \| 124,800 \| 52.19 \|  \| 31,200 \| 52.19 \|  \| \| Female \| 142,930 \| 47.81 \|  \| 114,344 \| 47.81 \|  \| 28,586 \| 47.81 \|  \| \| **Age (years)** \| 62.04 ± 18.90 \| \|  \| 61.81 ± 18.64 \| \|  \| 62.97 ± 19.21 \| \| 2.047E-5 \| \| **Age group (yrs)** \|  \|  \|  \|  \|  \|  \|  \|  \| .006 \| \| 20-29 \| 2,090 \| 0.70 \|  \| 1,655 \| 0.69 \|  \| 435 \| 0.73 \|  \| \| 30-39 \| 17,500 \| 5.85 \|  \| 13,989 \| 5.85 \|  \| 3,511 \| 5.87 \|  \| \| 40-49 \| 51,059 \| 17.08 \|  \| 40,903 \| 17.10 \|  \| 10,156 \| 16.99 \|  \| \| 50-59 \| 54,243 \| 18.15 \|  \| 43,601 \| 18.23 \|  \| 10,642 \| 17.80 \|  \| \| ≥60 \| 174,038 \| 58.22 \|  \| 138,996 \| 58.12 \|  \| 35,042 \| 58.61 \|  \| \| **Insured premium (NT$)** \|  \|  \|  \|  \|  \|  \|  \|  \| 1.264E-5 \| \| <18,000 \| 261,806 \| 87.58 \|  \| 209,465 \| 87.59 \|  \| 52,341 \| 87.55 \|  \| \| 18,000-34,999 \| 23,905 \| 8.00 \|  \| 19,127 \| 8.00 \|  \| 4,778 \| 7.99 \|  \| \| ≥35,000 \| 13,219 \| 4.42 \|  \| 10,552 \| 4.41 \|  \| 2,667 \| 4.46 \|  \| \| **Normal pregnancy** \| 37,698 \| 12.61 \|  \| 31,125 \| 13.02 \|  \| 6,573 \| 10.99 \| 2.570E-10 \| \| **Comorbidities** \|  \|  \|  \|  \|  \|  \|  \|  \|  \| \| CHF \| 1,820 \| 0.61 \|  \| 1,014 \| 0.42 \|  \| 806 \| 1.35 \| 4.352E-7 \| \| Gastric ulcer \| 955 \| 0.32 \|  \| 722 \| 0.30 \|  \| 233 \| 0.39 \| .186 \| \| Peptic ulcer \| 1,075 \| 0.36 \|  \| 796 \| 0.33 \|  \| 279 \| 0.47 \| .023 \| \| Gastrojejunal ulcer \| 641 \| 0.21 \|  \| 433 \| 0.18 \|  \| 208 \| 0.35 \| 1.067E-5 \| \| PE \| 214 \| 0.07 \|  \| 179 \| 0.07 \|  \| 35 \| 0.06 \| .787 \| \| GI hemorrhage \| 576 \| 0.19 \|  \| 447 \| 0.19 \|  \| 129 \| 0.22 \| .689 \| \| Budd-Chiari syndrome \| 8 \| 0.00 \|  \| 4 \| 0.00 \|  \| 4 \| 0.01 \| .995 \| \| Cerebral thrombosis \| 521 \| 0.17 \|  \| 372 \| 0.16 \|  \| 149 \| 0.25 \| .003 \| \| IHD \| 3,608 \| 1.21 \|  \| 2,583 \| 1.08 \|  \| 1,025 \| 1.71 \| .026 \| \| Vascular insufficiency of intestine \| 904 \| 0.30 \|  \| 697 \| 0.29 \|  \| 207 \| 0.35 \| .786 \| \| Obesity \| 303 \| 0.10 \|  \| 229 \| 0.10 \|  \| 74 \| 0.12 \| .677 \| \| HBV with hepatic coma \| 6,148 \| 2.06 \|  \| 4,813 \| 2.01 \|  \| 1,335 \| 2.23 \| .753 \| \| HBV without hepatic coma \| 33,180 \| 11.10 \|  \| 25,204 \| 10.54 \|  \| 7,976 \| 13.34 \| 6.289E-8 \| \| Malignant neoplasm of kidney/renal pelvis \| 7,747 \| 2.59 \|  \| 5,726 \| 2.39 \|  \| 2,021 \| 3.38 \| 3.121E-9 \| \| Acute glomerulonephritis/nephrotic syndrome \| 1,843 \| 0.62 \|  \| 1,334 \| 0.56 \|  \| 509 \| 0.85 \| 4.406E-6 \| \| Proteinuria \| 1,413 \| 0.47 \|  \| 1,068 \| 0.45 \|  \| 345 \| 0.58 \| .025 \| \| Gestational hypertension \| 2,463 \| 0.82 \|  \| 1,911 \| 0.80 \|  \| 552 \| 0.92 \| .068 \| \| Asthma \| 20,322 \| 6.80 \|  \| 16,652 \| 6.96 \|  \| 3,670 \| 6.14 \| .007 \| \| CCI_R \| 0.81 ± 1.11 \| \|  \| 0.80 ± 1.06 \| \|  \| 0.88 ± 1.23 \| \| 2.245E-6 \| \| **Medications** \|  \|  \|  \|  \|  \|  \|  \|  \|  \| \| Aspirin \| 42,333 \| 14.16 \|  \| 33,311 \| 13.93 \|  \| 9,022 \| 15.09 \| 7.562E-11 \| \| Celecoxib \| 34,666 \| 11.60 \|  \| 27,181 \| 11.37 \|  \| 7,485 \| 12.52 \| 5.601E-12 \| \| Itraconazole \| 15,280 \| 5.11 \|  \| 12,303 \| 5.14 \|  \| 2,977 \| 4.98 \| .007 \| \| Mebendazole \| 43,025 \| 14.39 \|  \| 34,890 \| 14.59 \|  \| 8,135 \| 13.61 \| .003 \| \| Leflunomide \| 20,418 \| 6.83 \|  \| 16,521 \| 6.91 \|  \| 3,897 \| 6.52 \| .184 \| \| Thalidomide \| 29,454 \| 9.85 \|  \| 23,292 \| 9.74 \|  \| 6,162 \| 10.31 \| 2.487E-6 \| \| Valproate \| 24,197 \| 8.09 \|  \| 18,862 \| 7.89 \|  \| 5,335 \| 8.92 \| 1.603E-5 \| \| Metformin \| 49,027 \| 16.40 \|  \| 39,030 \| 16.32 \|  \| 9,997 \| 16.72 \| .591 \| \| Auranofin \| 14,056 \| 4.70 \|  \| 10,378 \| 4.34 \|  \| 3,678 \| 6.15 \| 8.121E-7 \| \| Statins \| 41,379 \| 13.84 \|  \| 33,454 \| 13.99 \|  \| 7,925 \| 13.26 \| .067 \| \| Bisphosphonates \| 27,136 \| 9.08 \|  \| 21,906 \| 9.16 \|  \| 5,230 \| 8.75 \| 4.863E-5 \| \| Bromocriptine \| 29,507 \| 9.87 \|  \| 23,279 \| 9.73 \|  \| 6,228 \| 10.42 \| .003 \| \| Chlorprothixene \| 35,441 \| 11.86 \|  \| 28,306 \| 11.84 \|  \| 7,135 \| 11.93 \| .452 \| \| Clotrimazole \| 28,086 \| 9.40 \|  \| 22,184 \| 9.28 \|  \| 5,902 \| 9.87 \| .397 \| \| Quinacrine \| 25,125 \| 8.40 \|  \| 20,337 \| 8.50 \|  \| 4,788 \| 8.01 \| .688 \| \| Ivermectin \| 23,085 \| 7.72 \|  \| 17,972 \| 7.52 \|  \| 5,113 \| 8.55 \| 5.606E-6 \| \| Verteporfin \| 22,919 \| 7.67 \|  \| 18,994 \| 7.94 \|  \| 3,925 \| 6.57 \| 7.907E-7 \| \| Clarithromycin \| 12,084 \| 4.04 \|  \| 9,896 \| 4.14 \|  \| 2,188 \| 3.66 \| .002 \| \| Hydroxychloroquine \| 29,356 \| 9.82 \|  \| 23,897 \| 9.99 \|  \| 5,459 \| 9.13 \| .187 \| \| Tofacitinib \| 29,041 \| 9.71 \|  \| 23,014 \| 9.62 \|  \| 6,027 \| 10.08 \| 2.045E-5 \| \| Gefitinib \| 30,785 \| 10.30 \|  \| 25,113 \| 10.50 \|  \| 5,672 \| 9.49 \| 1.884E-6 \| \| Curcumin \| 14,817 \| 4.96 \|  \| 10,674 \| 4.46 \|  \| 4,143 \| 6.93 \| 6.672E-7 \| \| Chlorhexidine \| 16,016 \| 5.36 \|  \| 12,131 \| 5.07 \|  \| 3,885 \| 6.50 \| .010 \| \| Axitinib \| 11,614 \| 3.89 \|  \| 9,024 \| 3.77 \|  \| 2,590 \| 4.33 \| 2.920E-5 \| \| **Season of index date** \|  \|  \|  \|  \|  \|  \|  \|  \| 3.057E-6 \| \| Spring \| 63,270 \| 21.17 \|  \| 50,014 \| 20.91 \|  \| 13,256 \| 22.17 \|  \| \| Summer \| 74,721 \| 25.00 \|  \| 60,124 \| 25.14 \|  \| 14,597 \| 24.42 \|  \| \| Autumn \| 81,263 \| 27.18 \|  \| 65,284 \| 27.30 \|  \| 15,979 \| 26.73 \|  \| \| Winter \| 79,676 \| 26.65 \|  \| 63,722 \| 26.65 \|  \| 15,954 \| 26.69 \|  \| \| **Location** \|  \|  \|  \|  \|  \|  \|  \|  \| 6.255E-7 \| \| Northern Taiwan \| 112,598 \| 37.67 \|  \| 90,065 \| 37.66 \|  \| 22,533 \| 37.69 \|  \| \| Middle Taiwan \| 89,156 \| 29.83 \|  \| 71,989 \| 30.10 \|  \| 17,167 \| 28.71 \|  \| \| Southern Taiwan \| 54,615 \| 18.27 \|  \| 43,312 \| 18.11 \|  \| 11,303 \| 18.91 \|  \| \| Eastern Taiwan \| 37,142 \| 12.42 \|  \| 30,121 \| 12.60 \|  \| 7,021 \| 11.74 \|  \| \| Outlets islands \| 5,419 \| 1.81 \|  \| 3,657 \| 1.53 \|  \| 1,762 \| 2.95 \|  \| \| **Urbanization level** \|  \|  \|  \|  \|  \|  \|  \|  \| 5.254E-7 \| \| 1 (The highest) \| 111,358 \| 37.25 \|  \| 89,923 \| 37.60 \|  \| 21,435 \| 35.85 \|  \| \| 2 \| 97,653 \| 32.67 \|  \| 77,665 \| 32.48 \|  \| 19,988 \| 33.43 \|  \| \| 3 \| 38,140 \| 12.76 \|  \| 30,024 \| 12.55 \|  \| 8,116 \| 13.58 \|  \| \| 4 (The lowest) \| 51,779 \| 17.32 \|  \| 41,532 \| 17.37 \|  \| 10,247 \| 17.14 \|  \| \| **Levels of hospitals** \|  \|  \|  \|  \|  \|  \|  \|  \| 3.807E-6 \| \| Medical center \| 104,823 \| 35.07 \|  \| 84,066 \| 35.15 \|  \| 20,757 \| 34.72 \|  \| \| Regional hospital \| 102,375 \| 34.25 \|  \| 82,251 \| 34.39 \|  \| 20,124 \| 33.66 \|  \| \| Local hospital \| 91,732 \| 30.69 \|  \| 72,827 \| 30.45 \|  \| 18,905 \| 31.62 \|  \| | |
| ***P:* Chi-square / Fisher exact test on categorical variables and t-test on continuous variables**  Abbreviations: MDS, myelodysplastic syndromes; CHF, congestive heart failure; PE, pulmonary embolism; GI, gastrointestinal; IHD, ischemic heart disease; HBV, hepatitis B virus; CCI_R, Charlson comorbidity index_revised. | |
|  | |

| **Table J. Mortality Analysis of Patients with Hypertension by Prescription Duration of Hydralazine, 2000–2015** | | | | | | | | | | | | | | | | | | | | | | | | | | |
| --- | --- | --- | --- | --- | --- | --- | --- | --- | --- | --- | --- | --- | --- | --- | --- | --- | --- | --- | --- | --- | --- | --- | --- | --- | --- | --- |
| **Prescription Duration of Hydralazine** | | **≥180 days** | | | | | **<180 days** | | | | | | | | | **≥180 days vs. <180 days** (Reference) | | | | | | | | | | |
| **Mortality Analysis** | | **Events** | **PYs** | **Rate (per 10^5^ PYs)** | | | | **Events** | | **PYs** | **Rate (per 10^5^ PYs)** | | | | **Adjusted HR**‡ | | | | **95% CI** | | | | | ***P*** | |  |
| **Hematologic neoplasm-related mortality** | 153 | | 768,980.24 | | 19.90 | 661 | | | 2,897,605.18 | | | 22.81 | | 0.884 | | | | 0.632 | | | 1.238 | | .265 | | |  |
| Lymphosarcoma and reticulosarcoma | 3 | | 768,980.24 | | 0.39 | 11 | | | 2,897,605.18 | | | 0.38 | | 1.042 | | | | 0.744 | | | 459 | | .482 | | |  |
| Hodgkin's disease | 7 | | 768,980.24 | | 0.91 | 28 | | | 2,897,605.18 | | | 0.97 | | 0.955 | | | | 0.682 | | | 1.337 | | .384 | | |  |
| Other malignant neoplasms of lymphoid and histiocytic tissue | 20 | | 768,980.24 | | 2.60 | 103 | | | 2,897,605.18 | | | 3.55 | | 0.742 | | | | 0.530 | | | 1.039 | | .124 | | |  |
| Multiple myeloma and immunoproliferative neoplasms | 15 | | 768,980.24 | | 1.95 | 79 | | | 2,897,605.18 | | | 2.73 | | 0.725 | | | | 0.518 | | | 1.016 | | .097 | | |  |
| Lymphoid leukemia | 9 | | 768,980.24 | | 1.17 | 48 | | | 2,897,605.18 | | | 1.66 | | 0.716 | | | | 0.512 | | | 1.003 | | .058 | | |  |
| Myeloid leukemia | 11 | | 768,980.24 | | 1.43 | 56 | | | 2,897,605.18 | | | 1.93 | | 0.751 | | | | 0.536 | | | 1.051 | | .149 | | |  |
| Monocytic leukemia | 0 | | 768,980.24 | | 0.00 | 0 | | | 2,897,605.18 | | | 0.00 | | - | | | | - | | | - | | - | | |  |
| Other specified leukemia | 0 | | 768,980.24 | | 0.00 | 2 | | | 2,897,605.18 | | | 0.07 | | 0.000 | | | | - | | | - | | .989 | | |  |
| Leukemia of unspecified cell type | 21 | | 768,980.24 | | 2.73 | 72 | | | 2,897,605.18 | | | 2.48 | | 1.114 | | | | 0.796 | | | 1.560 | | .641 | | |  |
| Neoplasm of uncertain behavior | 10 | | 768,980.24 | | 1.30 | 53 | | | 2,897,605.18 | | | 1.83 | | 0.721 | | | | 0.515 | | | 1.009 | | .074 | | |  |
| MDS | 2 | | 768,980.24 | | 0.26 | 7 | | | 2,897,605.18 | | | 0.24 | 1.092 | | | | 0.780 | | | 1.528 | | .606 | | |  |  |
| Paraproteinemia | 25 | | 768,980.24 | | 3.25 | 96 | | | 2,897,605.18 | | | 3.31 | 0.995 | | | | 0.711 | | | 1.393 | | .399 | | |  |  |
| Other polycythemia | 30 | | 768,980.24 | | 3.90 | 106 | | | 2,897,605.18 | | | 3.66 | 1.081 | | | | 0.772 | | | 1.514 | | .572 | | |  |  |
| **All-cause mortality** | 6,789 | | 768,980.24 | | 882.86 | 24,121 | | | 2,897,605.18 | | | 832.45 | 1.075 | | | | 0.768 | | | 1.506 | | .536 | | |  |  |
| Abbreviations: PYs, person-years; CI, confidence interval; MDS, myelodysplastic syndrome;  ‡ Adjusted HR = adjusted hazard ratio; All variables controlled by the model (‡) include demographics (sex, age, insured premium, location, urbanization level, and level of hospital), comorbidities (congestive heart failure, pulmonary embolism, gastrointestinal hemorrhage, cerebral thrombosis, ischemic heart disease, vascular insufficiency of intestine, obesity, malignant neoplasm of kidney/renal pelvis, acute glomerulonephritis/nephrotic syndrome, proteinuria, gestational hypertension, and asthma), other variables (normal pregnancy and Charlson Comorbidity Index_Revised), and medications (aspirin, celecoxib, itraconazole, mebendazole, leflunomide, thalidomide, valproate, metformin, auranofin, statins [nystatin, lovastatin, pravastatin, simvastatin, atorvastatin, pitavastatin, rosuvastatin, cilastatin], bisphosphonates [alendronate and risedronate], bromocriptine, chlorprothixene, clotrimazole, quinacrine, ivermectin, verteporfin, clarithromycin, hydroxychloroquine, tofacitinib, gefitinib, curcumin, chlorhexidine, and axitinib). | | | | | | | | | | | | | | | | | | | | | | | | | | |

| **Table K.**  **Unadjusted (Crude) Hazard Ratios for Risk Factors Associated with Hematologic Neoplasm Development** | | | | | | | | | | | | | | | | | | | | | |
| --- | --- | --- | --- | --- | --- | --- | --- | --- | --- | --- | --- | --- | --- | --- | --- | --- | --- | --- | --- | --- | --- |
|  | **No Competing Risk Model** | | | | | | | | | **Fine and Gray's Competing Risk Model†** | | | | | | | | | | | |
| **Variables** | **Crude HR‡** | **95% CI** | | | | | | ***P*** | | **Crude sHR**§ | | | **95% CI** | | | | | ***P*** | | | |
| Hydralazine<180 days | Reference |  | | | | | | | | Reference | | |  | | | | | | | | |
| Hydralazine≥180 days | 0.782 | 0.663 | | | 0.930 | | | 0.015 | | 0.809 | | 0.677 | | | | 0.947 | | 0.024 | | | |
| **Sex** | | | | | | | | | | | | | | | | | | | | | |
| Male | 1.216 | | 0.906 | | | | 1.956 | | 0.250 | | 1.278 | | | 0.923 | | | 2.034 | | 0.311 | |  |
| Female | Reference | | |  | |  | |  | | Reference | | | | |  | | | | |  | |
| **Age group (yr)** | | | | | | | | | | | | | | | | | | | | | |
| 20-29 | Reference | | |  | |  | |  | | Reference | | | | | |  | | | |  | |
| 30-39 | 1.188 | | | 0.669 | | 1.446 | | 0.345 | | 1.333 | | 0.395 | | | 1.964 | | | 0.604 | | | |
| 40-49 | 1.151 | | | 0.536 | | 1.376 | | 0.497 | | 1.225 | | 0.236 | | | 1.795 | | | 0.760 | | | |
| 50-59 | 1.148 | | | 0.549 | | 1.401 | | 0.450 | | 1.236 | | 0.243 | | | 1.818 | | | 0.736 | | | |
| ≥60 | 1.203 | | | 0.685 | | 1.456 | | 0.315 | | 1.355 | | 0.403 | | | 1.994 | | | 0.597 | | | |
| **Insured premium (NTD)** | | | | | | | | | | | | | | | | | | | | | |
| <18,000 | Reference | | |  | |  | |  | | Reference | | | | |  | | |  | | | |
| 18,000-34,999 | 1.097 | | | 0.737 | | 1.795 | | 0.266 | | 1.130 | | 0.761 | | | 1.821 | | | 0.269 | | | |
| ≥35,000 | 0.812 | | | 0.491 | | 1.230 | | 0.504 | | 0.917 | | 0.507 | | | 1.296 | | | 0.493 | | | |
| **Normal pregnancy** | 1.516 | | | 0.976 | | 2.394 | | 0.082 | | 1.787 | | 1.007 | | | 2.755 | | | 0.047 | | | |
| **Comorbidities**(Reference: Without) | | | | | | | | | | | | | | | | | | | | | |
| CHF | 0.980 | | | 0.720 | | 1.229 | | 0.281 | | 1.307 | | 1.075 | | | 1.633 | | | 0.013 | | | |
| PE | 1.172 | | | 0.870 | | 1.491 | | 0.135 | | 1.440 | | 1.021 | | | 1.716 | | | 0.040 | | | |
| GI hemorrhage | 1.240 | | | 0.718 | | 1.677 | | 0.292 | | 1.536 | | 1.030 | | | 2.276 | | | 0.035 | | | |
| Cerebral thrombosis | 2.821 | | | 0.126 | | 6.008 | | 0.893 | | 3.074 | | 0.187 | | | 6.464 | | | 0.823 | | | |
| IHD | 1.062 | | | 0.561 | | 1.184 | | 0.462 | | 1.162 | | 0.760 | | | 1.296 | | | 0.359 | | | |
| Vascular insufficiency of intestine | 1.684 | | | 1.192 | | 2.123 | | < 0.001 | | 2.002 | | 1.531 | | | 2.465 | | | < 0.001 | | | |
| Obesity | 1.195 | | | 1.047 | | 1.709 | | 0.026 | | 1.648 | | 1.191 | | | 2.130 | | | < 0.001 | | | |
| HBV with hepatic coma | 1.467 | | | 0.217 | | 2.115 | | 0.792 | | 1.705 | | 0.381 | | | 2.902 | | | 0.620 | | | |
| HBV without hepatic coma | 2.720 | | | 1.788 | | 3.616 | | < 0.001 | | 3.102 | | 1.828 | | | 3.725 | | | < 0.001 | | | |
| Malignant neoplasm of kidney/renal pelvis | 2.155 | | | 1.376 | | 3.087 | | < 0.001 | | 2.314 | | 1.471 | | | 3.096 | | | < 0.001 | | | |
| Acute glomerulonephritis/  Nephrotic syndrome | 1.661 | | | 1.089 | | 2.022 | | 0.005 | | 1.746 | | 1.101 | | | 2.070 | | | < 0.001 | | | |
| Proteinuria | 1.684 | | | 1.150 | | 2.222 | | < 0.001 | | 2.268 | | 1.529 | | | 4.074 | | | < 0.001 | | | |
| Gestational hypertension | 1.222 | | | 0.843 | | 1.641 | | 0.154 | | 1.341 | | 0.928 | | | 1.714 | | | 0.092 | | | |
| Asthma | 0.917 | | | 0.507 | | 1.187 | | 0.493 | | 0.847 | | 0.485 | | | 1.096 | | | 0.562 | | | |
| CCI_R | 1.554 | | | 0.823 | | 2.179 | | 0.177 | | 1.656 | | 0.876 | | | 2.261 | | | 0.125 | | | |
| **Medications** (Reference: Without) | | | | | | | | | | | | | | | | | | | | | |
| Aspirin | 1.616 | | | 1.094 | | 2.129 | | 0.003 | | 1.696 | | 1.117 | | | 2.211 | | | < 0.001 | | | |
| Celecoxib | 1.552 | | | 1.087 | | 1.914 | | 0.006 | | 1.649 | | 1.180 | | | 2.004 | | | < 0.001 | | | |
| Itraconazole | 0.812 | | | 0.319 | | 1.714 | | 0.684 | | 0.951 | | 0.391 | | | 1.752 | | | 0.609 | | | |
| Mebendazole | 0.597 | | | 0.183 | | 0.971 | | 0.813 | | 0.714 | | 0.290 | | | 0.992 | | | 0.704 | | | |
| Leflunomide | 1.323 | | | 0.843 | | 1.622 | | 0.257 | | 1.421 | | 0.884 | | | 1.712 | | | 0.105 | | | |
| Thalidomide | 1.605 | | | 1.153 | | 2.265 | | < 0.001 | | 1.894 | | 1.212 | | | 2.557 | | | < 0.001 | | | |
| Valproate | 1.117 | | | 0.369 | | 1.397 | | 0.632 | | 1.513 | | 0.713 | | | 2.260 | | | 0.284 | | | |
| Metformin | 0.891 | | | 0.681 | | 1.075 | | 0.309 | | 0.954 | | 0.739 | | | 1.185 | | | 0.271 | | | |
| Auranofin | 1.193 | | | 0.709 | | 1.802 | | 0.295 | | 1.401 | | 0.859 | | | 1.974 | | | 0.146 | | | |
| Statins | 1.301 | | | 0.829 | | 1.936 | | 0.183 | | 1.675 | | 1.019 | | | 2.238 | | | 0.040 | | | |
| Bisphosphonates | 1.134 | | | 0.604 | | 1.716 | | 0.392 | | 1.262 | | 0.633 | | | 1.795 | | | 0.385 | | | |
| Bromocriptine | 1.296 | | | 0.664 | | 1.966 | | 0.345 | | 1.335 | | 0.669 | | | 2.070 | | | 0.333 | | | |
| Chlorprothixene | 1.176 | | | 0.459 | | 2.048 | | 0.542 | | 1.277 | | 0.489 | | | 2.105 | | | 0.513 | | | |
| Clotrimazole | 1.945 | | | 0.606 | | 2.426 | | 0.393 | | 2.013 | | 0.642 | | | 2.473 | | | 0.358 | | | |
| Quinacrine | 1.063 | | | 0.727 | | 1.920 | | 0.277 | | 1.104 | | 0.736 | | | 2.059 | | | 0.270 | | | |
| Ivermectin | 1.790 | | | 0.395 | | 2.976 | | 0.604 | | 1.798 | | 0.407 | | | 3.008 | | | 0.595 | | | |
| Verteporfin | 1.520 | | | 0.906 | | 2.071 | | 0.097 | | 1.541 | | 0.912 | | | 2.210 | | | 0.088 | | | |
| Clarithromycin | 1.238 | | | 0.133 | | 1.643 | | 0.864 | | 1.317 | | 0.179 | | | 1.687 | | | 0.830 | | | |
| Hydroxychloroquine | 1.011 | | | 0.259 | | 1.311 | | 0.744 | | 1.057 | | 0.588 | | | 1.395 | | | 0.423 | | | |
| Tofacitinib | 1.369 | | | 0.809 | | 1.867 | | 0.101 | | 1.383 | | 0.815 | | | 1.896 | | | 0.175 | | | |
| Gefitinib | 1.153 | | | 0.551 | | 1.737 | | 0.492 | | 1.196 | | 0.560 | | | 1.439 | | | 0.440 | | | |
| Curcumin | 1.116 | | | 0.680 | | 1.386 | | 0.323 | | 1.156 | | 0.678 | | | 1.400 | | | 0.315 | | | |
| Chlorhexidine | 1.330 | | | 0.897 | | 1.565 | | 0.109 | | 1.353 | | 0.910 | | | 1.631 | | | 0.093 | | | |
| Axitinib | 1.337 | | | 1.015 | | 1.581 | | 0.042 | | 1.359 | | 1.041 | | | 1.658 | | | 0.030 | | | |
| **Season of index date** |  | | | | | | | | | | | | | | | | | | | | |
| Spring | Reference | | |  | |  | |  | | Reference | | | | |  | | |  | | | |
| Summer | 0.764 | | | 0.500 | | 1.229 | | 0.402 | | 0.936 | | 0.734 | | | 1.305 | | | 0.267 | | | |
| Autumn | 0.590 | | | 0.411 | | 1.165 | | 0.598 | | 0.811 | | 0.660 | | | 1.268 | | | 0.331 | | | |
| Winter | 0.847 | | | 0.674 | | 1.472 | | 0.329 | | 0.913 | | 0.688 | | | 1.585 | | | 0.314 | | | |
| **Urbanization level** | | | | | | | | | | | | | | | | | | | | | |
| 1 (The highest) | 1.391 | | | 0.838 | | 1.928 | | 0.169 | | 1.432 | | 0.851 | | | 1.934 | | | 0.142 | | | |
| 2 | 1.261 | | | 0.718 | | 1.851 | | 0.283 | | 1.323 | | 0.760 | | | 1.893 | | | 0.245 | | | |
| 3 | 1.159 | | | 0.631 | | 1.813 | | 0.392 | | 1.227 | | 0.689 | | | 1.836 | | | 0.313 | | | |
| 4 (The lowest) | Reference | | |  | |  | |  | | Reference | | | | |  | | |  | | | |
| **Levels of hospitals** | | | | | | | | | | | | | | | | | | | | | |
| Medical center | 2.534 | | | 2.095 | | 3.012 | | < 0.001 | | 2.534 | | 2.095 | | | 3.012 | | | < 0.001 | | | |
| Regional hospital | 2.137 | | | 1.669 | | 2.566 | | < 0.001 | | 2.137 | | 1.669 | | | 2.566 | | | < 0.001 | | | |
| Local hospital | Reference | | |  | |  | |  | | Reference | | | | |  | | |  | | | |
| Abbreviations: NTD = New Taiwan dollar; CHF = congestive heart failure; PE = pulmonary embolism; GI = gastrointestinal; IHD = ischemic heart disease; HBV = hepatitis B virus; CCI_R = Charlson Comorbidity Index_Revised; HR = hazard ratio; sHR = subdistribution hazard ratio; CI = confidence interval †Competing variable was all-cause mortality. | | | | | | | | | | | | | | | | | | | | | |

| **Table L. Unadjusted (Crude) Hazard Ratios for Hematologic Neoplasm Development, Stratified by Prescription Duration of Hydralazine** | | | | | | | | | |
| --- | --- | --- | --- | --- | --- | --- | --- | --- | --- |
| **Subgroups of Hematologic Neoplasms** | **Prescription Duration of Hydralazine** | **Population** | **Events** | **PYs** | **Rate (per 10^5^ PYs)** | **Crude HR** | **95% CI** | | ***P*** |
| Overall | < 180 days | 239,144 | 4,544 | 2,834,197.06 | 160.33 | Reference |  |  |  |
|  | ≥180 days | 59,786 | 757 | 716,983.56 | 105.58 | 0.782 | 0.663 | 0.930 | 0.015 |
|  | 180-350 days | 19,868 | 294 | 238,267.67 | 123.39 | 0.907 | 0.754 | 1.139 | 0.246 |
|  | 351-667 days | 19,975 | 245 | 239,805.11 | 102.17 | 0.747 | 0.607 | 0.937 | 0.018 |
|  | ≥668 days | 19,943 | 218 | 238,910.78 | 91.25 | 0.663 | 0.539 | 0.833 | < 0.001 |
| Lymphosarcoma and reticulosarcoma | < 180 days | 239,144 | 20 | 2,834,197.06 | 0.71 | Reference |  |  |  |
|  | ≥ 180 days | 59,786 | 6 | 716,983.56 | 0.84 | 1.537 | 0.996 | 1.930 | 0.053 |
|  | 180-350 days | 19,868 | 3 | 238,267.67 | 1.26 | 2.315 | 1.067 | 2.907 | 0.017 |
|  | 351-667 days | 19,975 | 2 | 239,805.11 | 0.83 | 1.147 | 0.931 | 1.439 | 0.067 |
|  | ≥668 days | 19,943 | 1 | 238,910.78 | 0.42 | 0.915 | 0.539 | 1.291 | 0.471 |
| Hodgkin's disease | < 180 days | 239,144 | 61 | 2,834,197.06 | 2.15 | Reference |  |  |  |
|  | ≥180 days | 59,786 | 18 | 716,983.56 | 2.51 | 1.301 | 0.992 | 1.633 | 0.058 |
|  | 180-350 days | 19,868 | 9 | 238,267.67 | 3.78 | 2.138 | 1.128 | 2.683 | < 0.001 |
|  | 351-667 days | 19,975 | 5 | 239,805.11 | 2.09 | 1.015 | 0.859 | 1.328 | 0.141 |
|  | ≥668 days | 19,943 | 4 | 238,910.78 | 1.67 | 0.710 | 0.577 | 0.892 | < 0.001 |
| Other malignant neoplasms of lymphoid and histiocytic tissue | < 180 days | 239,144 | 541 | 2,834,197.06 | 19.09 | Reference |  |  |  |
|  | ≥180 days | 59,786 | 68 | 716,983.56 | 9.48 | 0.572 | 0.466 | 0.720 | < 0.001 |
|  | 180-350 days | 19,868 | 28 | 238,267.67 | 11.75 | 0.741 | 0.602 | 0.929 | 0.015 |
|  | 351-667 days | 19,975 | 21 | 239,805.11 | 8.76 | 0.529 | 0.430 | 0.665 | < 0.001 |
|  | ≥668 days | 19,943 | 19 | 238,910.78 | 7.95 | 0.451 | 0.366 | 0.565 | < 0.001 |
| Multiple myeloma and immunoproliferative neoplasms | < 180 days | 239,144 | 369 | 2,834,197.06 | 13.02 | Reference |  |  |  |
|  | ≥180 days | 59,786 | 53 | 716,983.56 | 7.39 | 0.613 | 0.499 | 0.770 | < 0.001 |
|  | 180-350 days | 19,868 | 19 | 238,267.67 | 7.97 | 0.673 | 0.546 | 0.844 | < 0.001 |
|  | 351-667 days | 19,975 | 18 | 239,805.11 | 7.51 | 0.610 | 0.496 | 0.766 | < 0.001 |
|  | ≥668 days | 19,943 | 16 | 238,910.78 | 6.70 | 0.558 | 0.454 | 0.701 | < 0.001 |
| Lymphoid leukemia | < 180 days | 239,144 | 158 | 2,834,197.06 | 5.57 | Reference |  |  |  |
|  | ≥180 days | 59,786 | 25 | 716,983.56 | 3.49 | 0.899 | 0.607 | 1.022 | 0.394 |
|  | 180-350 days | 19,868 | 13 | 238,267.67 | 5.46 | 1.021 | 0.912 | 1.409 | 0.088 |
|  | 351-667 days | 19,975 | 7 | 239,805.11 | 2.92 | 0.556 | 0.452 | 0.698 | < 0.001 |
|  | ≥668 days | 19,943 | 5 | 238,910.78 | 2.09 | 0.420 | 0.341 | 0.527 | < 0.001 |
| Myeloid leukemia | < 180 days | 239,144 | 237 | 2,834,197.06 | 8.36 | Reference |  |  |  |
|  | ≥180 days | 59,786 | 37 | 716,983.56 | 5.16 | 0.716 | 0.582 | 0.900 | 0.001 |
|  | 180-350 days | 19,868 | 14 | 238,267.67 | 5.88 | 0.845 | 0.687 | 1.061 | 0.324 |
|  | 351-667 days | 19,975 | 12 | 239,805.11 | 5.00 | 0.744 | 0.605 | 0.933 | 0.017 |
|  | ≥668 days | 19,943 | 11 | 238,910.78 | 4.60 | 0.561 | 0.457 | 0.705 | < 0.001 |
| Other specified leukemia | < 180 days | 239,144 | 17 | 2,834,197.06 | 0.60 | Reference |  |  |  |
|  | ≥180 days | 59,786 | 3 | 716,983.56 | 0.42 | 0.922 | 0.750 | 1.158 | 0.251 |
|  | 180-350 days | 19,868 | 2 | 238,267.67 | 0.84 | 1.389 | 0.942 | 1.744 | 0.068 |
|  | 351-667 days | 19,975 | 1 | 239,805.11 | 0.42 | 1.051 | 0.897 | 1.406 | 0.107 |
|  | ≥668 days | 19,943 | 0 | 238,910.78 | 0.00 | 0.000 | - | - | 0.999 |
| Leukemia of unspecified cell type | < 180 days | 239,144 | 542 | 2,834,197.06 | 19.12 | Reference |  |  |  |
|  | ≥180 days | 59,786 | 90 | 716,983.56 | 12.55 | 0.778 | 0.632 | 0.977 | 0.037 |
|  | 180-350 days | 19,868 | 32 | 238,267.67 | 13.43 | 0.823 | 0.669 | 1.032 | 0.333 |
|  | 351-667 days | 19,975 | 30 | 239,805.11 | 12.51 | 0.773 | 0.628 | 0.971 | 0.035 |
|  | ≥668 days | 19,943 | 28 | 238,910.78 | 11.72 | 0.738 | 0.600 | 0.926 | 0.013 |
| Neoplasm of uncertain behavior | < 180 days | 239,144 | 653 | 2,834,197.06 | 23.04 | Reference |  |  |  |
|  | ≥180 days | 59,786 | 101 | 716,983.56 | 14.09 | 0.741 | 0.602 | 0.930 | 0.015 |
|  | 180-350 days | 19,868 | 45 | 238,267.67 | 18.89 | 0.981 | 0.797 | 1.231 | 0.205 |
|  | 351-667 days | 19,975 | 32 | 239,805.11 | 13.34 | 0.703 | 0.572 | 0.882 | < 0.001 |
|  | ≥668 days | 19,943 | 24 | 238,910.78 | 10.05 | 0.540 | 0.438 | 0.677 | < 0.001 |
| MDS | < 180 days | 239,144 | 21 | 2,834,197.06 | 0.74 | Reference |  |  |  |
|  | ≥180 days | 59,786 | 5 | 716,983.56 | 0.70 | 0.883 | 0.641 | 1.292 | 0.356 |
|  | 180-350 days | 19,868 | 2 | 238,267.67 | 0.84 | 1.220 | 0.806 | 1.748 | 0.192 |
|  | 351-667 days | 19,975 | 2 | 239,805.11 | 0.83 | 1.184 | 0.801 | 1.685 | 0.199 |
|  | ≥668 days | 19,943 | 1 | 238,910.78 | 0.42 | 0.591 | 0.325 | 0.815 | < 0.001 |
| Paraproteinemia | < 180 days | 239,144 | 203 | 2,834,197.06 | 7.16 | Reference |  |  |  |
|  | ≥180 days | 59,786 | 48 | 716,983.56 | 6.69 | 1.015 | 0.872 | 1.346 | 0.139 |
|  | 180-350 days | 19,868 | 24 | 238,267.67 | 10.07 | 1.616 | 1.007 | 2.028 | 0.046 |
|  | 351-667 days | 19,975 | 14 | 239,805.11 | 5.84 | 0.959 | 0.780 | 1.205 | 0.222 |
|  | ≥668 days | 19,943 | 10 | 238,910.78 | 4.19 | 0.644 | 0.524 | 0.809 | < 0.001 |
| Other polycythemia | < 180 days | 239,144 | 1,722 | 2,834,197.06 | 60.76 | Reference |  |  |  |
|  | ≥180 days | 59,786 | 303 | 716,983.56 | 42.26 | 0.810 | 0.659 | 0.930 | 0.015 |
|  | 180-350 days | 19,868 | 103 | 238,267.67 | 43.23 | 0.827 | 0.672 | 0.958 | 0.029 |
|  | 351-667 days | 19,975 | 101 | 239,805.11 | 42.12 | 0.805 | 0.655 | 0.886 | < 0.001 |
|  | ≥668 days | 19,943 | 99 | 238,910.78 | 41.44 | 0.798 | 0.649 | 0.853 | < 0.001 |
| Abbreviations: PYs = person-years; HR = hazard ratio; CI = confidence interval; MDS = myelodysplastic syndrome  The subgroup of monocytic leukemia is not listed due to the lack of events. | | | | | | | | | |

| **Table M.**  **Unadjusted (Crude) Hazard Ratios for Sensitivity Analysis of Hematologic Neoplasm Development** | | | | | | | | | | | | | |
| --- | --- | --- | --- | --- | --- | --- | --- | --- | --- | --- | --- | --- | --- |
|  | | | | |  | **No competing risk model** | | |  | **Fine and Gray’s competing risk model*** | | | |
| **Sensitivity Analysis** | **Prescription Duration of Hydralazine** | **Populations** | **Events** | **PYs** | **Rate (per 10^5^ PYs)** | **Crude HR** | **95% CI** | | ***P*** | **Crude sHR** | **95% CI** | | ***P*** |
| Overall | < 180 days | 239,144 | 4,544 | 2,834,197.06 | 160.33 | Reference |  |  |  | Reference |  |  |  |
|  | ≥ 180 days | 59,786 | 757 | 716,983.56 | 105.58 | 0.782 | 0.663 | 0.930 | 0.015 | 0.809 | 0.677 | 0.947 | 0.024 |
|  | 180-350 days | 19,868 | 294 | 238,267.67 | 123.39 | 0.907 | 0.754 | 1.139 | 0.246 | 0.940 | 0.778 | 1.176 | 0.235 |
|  | 351-667 days | 19,975 | 245 | 239,805.11 | 102.17 | 0.747 | 0.607 | 0.937 | 0.018 | 0.773 | 0.627 | 0.970 | 0.035 |
|  | ≥668 days | 19,943 | 218 | 238,910.78 | 91.25 | 0.663 | 0.539 | 0.833 | < 0.001 | 0.683 | 0.560 | 0.862 | < 0.001 |
| In the first year excluded | < 180 days | 239,144 | 4,260 | 2,657,043.24 | 160.33 | Reference |  |  |  | Reference |  |  |  |
|  | ≥ 180 days | 59,786 | 711 | 672,174.25 | 105.78 | 0.779 | 0.633 | 1.008 | 0.057 | 0.807 | 0.655 | 1.016 | 0.063 |
|  | 180-350 days | 19,868 | 276 | 223,378.13 | 123.56 | 0.910 | 0.740 | 1.144 | 0.253 | 0.942 | 0.767 | 1.190 | 0.239 |
|  | 351-667 days | 19,975 | 235 | 224,897.20 | 104.49 | 0.755 | 0.614 | 0.949 | 0.025 | 0.782 | 0.632 | 0.982 | 0.041 |
|  | ≥668 days | 19,943 | 200 | 223,898.92 | 89.33 | 0.669 | 0.546 | 0.842 | < 0.001 | 0.691 | 0.563 | 0.869 | < 0.001 |
| In the first 5 years excluded | < 180 days | 239,144 | 3,115 | 1,948,522.27 | 159.86 | Reference |  |  |  | Reference |  |  |  |
|  | ≥ 180 days | 59,786 | 517 | 492,464.35 | 104.98 | 0.791 | 0.645 | 0.992 | 0.046 | 0.819 | 0.669 | 0.985 | 0.043 |
|  | 180-350 days | 19,868 | 203 | 163,798.24 | 123.93 | 0.924 | 0.734 | 1.133 | 0.268 | 0.959 | 0.754 | 1.174 | 0.246 |
|  | 351-667 days | 19,975 | 166 | 164,863.25 | 100.69 | 0.764 | 0.622 | 0.961 | 0.030 | 0.790 | 0.641 | 0.981 | 0.041 |
|  | ≥668 days | 19,943 | 148 | 163,802.86 | 90.35 | 0.683 | 0.556 | 0.859 | < 0.001 | 0.702 | 0.573 | 0.890 | < 0.001 |
| Abbreviations: PYs = person-years; HR = hazard ratio; CI = confidence interval; sHR = subdistribution hazard ratio | | | | | | | | | | | | | |
| * Competing variable was all-cause mortality. | | | | | | | | | | | | | |

| **Table N. Unadjusted (Crude) Subdistribution Hazard Ratios for First-Event and Multiple-Event Models**† | | | | | | | | | | | | | | | | | | | | | | | |
| --- | --- | --- | --- | --- | --- | --- | --- | --- | --- | --- | --- | --- | --- | --- | --- | --- | --- | --- | --- | --- | --- | --- | --- |
| **Subgroups of Hematologic Neoplasms** | **Prescription Duration of Hydralazine** | **Population** | **First-Event Model** | | | | | | | | **Multiple-Event Model** | | | | | | | | | | | | |
|  |  |  | **Events** | **Crude sHR**§ | | | **95% CI** | | | ***P*** | **Events** | **Crude sHR**§ | **95% CI** | | | | | | | ***P*** | | | |
| **Overall (Hematologic neoplasms)** | <180 days | 239,144 | 4,544 | Reference | | |  | |  |  | - | - | - | | - | | | | | - | | | |
|  | ≥180 days | 59,786 | 757 | 0.809 | | | 0.677 | | 0.947 | 0.024 | - | - | - | | - | | | | | - | | | |
|  | 180-350 days | 19,868 | 294 | 0.940 | | | 0.778 | | 1.176 | 0.235 | - | - | - | | - | | | | | - | | | |
|  | 351-667 days | 19,975 | 245 | 0.773 | | | 0.627 | | 0.970 | 0.035 | - | - | - | | - | | | | | - | | | |
|  | ≥668 days | 19,943 | 218 | 0.683 | | | 0.560 | | 0.862 | < 0.001 | - | - | - | | - | | | | | - | | | |
| **Lymphosarcoma and reticulosarcoma** | <180 days | 239,144 | 20 | Reference | | |  | |  |  | 33 | Reference |  | |  | | | | |  | | | |
|  | ≥180 days | 59,786 | 6 | 1.556 | | | 0.969 | | 1.955 | 0.068 | 24 | 1.516 | 1.014 | | 1.904 | | | | | 0.043 | | | |
|  | 180-350 days | 19,868 | 3 | 2.348 | | | 1.082 | | 2.952 | 0.009 | 12 | 2.288 | 1.054 | | 2.869 | | | | | 0.023 | | | |
|  | 351-667 days | 19,975 | 2 | 1.163 | | | 0.944 | | 1.458 | 0.076 | 8 | 1.126 | 0.919 | | 1.411 | | | | | 0.084 | | | |
|  | ≥668 days | 19,943 | 1 | 0.926 | | | 0.546 | | 1.308 | 0.452 | 4 | 1.139 | 0.924 | | 1.420 | | | | | 0.079 | | | |
| **Hodgkin's disease** | <180 days | 239,144 | 61 | Reference | | |  | |  |  | 65 | Reference |  | |  | | | | |  | | | |
|  | ≥180 days | 59,786 | 18 | 1.317 | | | 1.006 | | 1.654 | 0.047 | 33 | 1.275 | 0.981 | | 1.610 | | | | | 0.059 | | | |
|  | 180-350 days | 19,868 | 9 | 2.165 | | | 1.143 | | 2.721 | < 0.001 | 17 | 2.108 | 1.077 | | 2.637 | | | | | 0.012 | | | |
|  | 351-667 days | 19,975 | 5 | 1.028 | | | 0.870 | | 1.341 | 0.239 | 10 | 1.044 | 0.834 | | 1.267 | | | | | 0.168 | | | |
|  | ≥668 days | 19,943 | 4 | 0.719 | | | 0.584 | | 0.903 | 0.002 | 6 | 0.698 | 0.549 | | 0.863 | | | | | < 0.001 | | | |
| **Other malignant neoplasms of lymphoid and histiocytic tissue** | <180 days | 239,144 | 541 | Reference | | |  | |  |  | 567 | Reference |  | |  | | | | |  | | | |
|  | ≥180 days | 59,786 | 68 | 0.580 | | | 0.472 | | 0.729 | < 0.001 | 111 | 0.565 | 0.457 | | 0.712 | | | | | < 0.001 | | | |
|  | 180-350 days | 19,868 | 28 | 0.751 | | | 0.611 | | 0.943 | 0.022 | 44 | 0.730 | 0.589 | | 0.916 | | | | | 0.008 | | | |
|  | 351-667 days | 19,975 | 21 | 0.538 | | | 0.437 | | 0.674 | < 0.001 | 39 | 0.521 | 0.422 | | 0.651 | | | | | < 0.001 | | | |
|  | ≥668 days | 19,943 | 19 | 0.460 | | | 0.371 | | 0.575 | < 0.001 | 28 | 0.444 | 0.356 | | 0.566 | | | | | < 0.001 | | | |
| **Multiple myeloma and immunoproliferative neoplasms** | <180 days | 239,144 | 369 | Reference | | |  | |  |  | 387 | Reference |  | | | | | |  | | | |  |
|  | ≥180 days | 59,786 | 53 | 0.632 | | | 0.504 | | 0.781 | < 0.001 | 65 | 0.622 | 0.505 | 0.776 | | | | | | | < 0.001 | | |
|  | 180-350 days | 19,868 | 19 | 0.684 | | | 0.556 | | 0.860 | < 0.001 | 24 | 0.681 | 0.555 | 0.858 | | | | | | | < 0.001 | | |
|  | 351-667 days | 19,975 | 18 | 0.619 | | | 0.503 | 0.778 | | < 0.001 | 21 | 0.619 | 0.500 | | 0.769 | | | | | | < 0.001 | | |
|  | ≥668 days | 19,943 | 16 | 0.566 | | | 0.460 | 0.708 | | < 0.001 | 20 | 0.564 | 0.462 | | 0.708 | | | | | | < 0.001 | | |
| **Lymphoid leukemia** | <180 days | 239,144 | 158 | Reference | | |  |  | |  | 160 | Reference |  | |  | | | | |  | | | |
|  | ≥180 days | 59,786 | 25 | 0.911 | | | 0.617 | 1.035 | | 0.385 | 38 | 0.914 | 0.618 | | 1.036 | | | | | 0.384 | | | |
|  | 180-350 days | 19,868 | 13 | 1.035 | | | 0.924 | 1.428 | | 0.074 | 15 | 1.140 | 0.924 | | 1.431 | | | | | 0.076 | | | |
|  | 351-667 days | 19,975 | 7 | 0.563 | | | 0.459 | 0.707 | | < 0.001 | 13 | 0.564 | 0.465 | | 0.718 | | | | | < 0.001 | | | |
|  | ≥668 days | 19,943 | 5 | 0.424 | | | 0.347 | 0.530 | | < 0.001 | 10 | 0.427 | 0.349 | | 0.537 | | | | | < 0.001 | | | |
| **Myeloid leukemia** | <180 days | 239,144 | 237 | Reference | | |  |  | |  | 256 | Reference |  | |  | | | | |  | | | |
|  | ≥180 days | 59,786 | 37 | 0.725 | | | 0.591 | 0.910 | | 0.005 | 70 | 0.699 | 0.555 | | 0.875 | | | | | < 0.001 | | | |
|  | 180-350 days | 19,868 | 14 | 0.857 | | | 0.699 | 1.073 | | 0.301 | 30 | 0.836 | 0.668 | | 1.025 | | | | | 0.339 | | | |
|  | 351-667 days | 19,975 | 12 | 0.754 | | 0.613 | | | 0.945 | 0.022 | 22 | 0.730 | 0.582 | 0.905 | | | | | | 0.003 | | | |
|  | ≥668 days | 19,943 | 11 | 0.569 | | 0.464 | | | 0.714 | < 0.001 | 18 | 0.552 | 0.436 | 0.689 | | | | | | < 0.001 | | | |
| **Other specified leukemia** | <180 days | 239,144 | 17 | Reference | |  | | |  |  | 27 | Reference |  | |  | | | | | |  | | |
|  | ≥180 days | 59,786 | 3 | 0.935 | | 0.753 | | | 1.180 | 0.248 | 23 | 0.911 | 0.740 | | 1.143 | | | | | | 0.257 | | |
|  | 180-350 days | 19,868 | 2 | 1.407 | | 0.953 | | | 1.768 | 0.085 | 14 | 1.371 | 1.005 | | 1.723 | | | | | | 0.058 | | |
|  | 351-667 days | 19,975 | 1 | 1.065 | | 0.908 | | | 1.427 | 0.111 | 6 | 1.358 | 0.885 | | 1.706 | | | | | | 0.129 | | |
|  | ≥668 days | 19,943 | 0 | 0.000 | | - | | | - | 0.999 | 3 | 0.699 | 0.250 | | 0.886 | | | | | | < 0.001 | | |
| **Leukemia of unspecified cell type** | <180 days | 239,144 | 542 | Reference |  | | | |  |  | 597 | Reference |  | |  | | | | | | |  | |
|  | ≥180 days | 59,786 | 90 | 0.787 | 0.640 | | | | 0.987 | 0.042 | 163 | 0.787 | 0.641 | | 0.990 | | | | | | | 0.045 | |
|  | 180-350 days | 19,868 | 32 | 0.834 | 0.676 | | | | 1.045 | 0.335 | 66 | 0.832 | 0.677 | | 1.045 | | | | | | | 0.324 | |
|  | 351-667 days | 19,975 | 30 | 0.782 | 0.637 | | | | 0.974 | 0.037 | 57 | 0.783 | 0.636 | | 0.978 | | | | | | | 0.039 | |
|  | ≥668 days | 19,943 | 28 | 0.749 | 0.611 | | | | 0.937 | 0.018 | 40 | 0.746 | 0.609 | | 0.943 | | | | | | | 0.021 | |
| **Neoplasm of uncertain behavior** | <180 days | 239,144 | 653 | Reference | |  | | |  |  | 673 | Reference |  | | |  | | | | | |  | |
|  | ≥180 days | 59,786 | 101 | 0.751 | | 0.610 | | | 0.943 | 0.021 | 138 | 0.732 | 0.596 | | | 0.917 | | | | | | 0.007 | |
|  | 180-350 days | 19,868 | 45 | 0.994 | | 0.806 | | | 1.258 | 0.193 | 55 | 0.968 | 0.784 | | 1.200 | | | | | 0.218 | | | |
|  | 351-667 days | 19,975 | 32 | 0.711 | | 0.581 | | | 0.894 | < 0.001 | 48 | 0.694 | 0.563 | | 0.872 | | | | | < 0.001 | | | |
|  | ≥668 days | 19,943 | 24 | 0.546 | | 0.444 | | | 0.691 | < 0.001 | 35 | 0.538 | 0.432 | | 0.671 | | | | | < 0.001 | | | |
| **MDS** | <180 days | 239,144 | 21 | Reference | |  | | |  |  | 24 | Reference |  | | |  | | | |  | | | |
|  | ≥180 days | 59,786 | 5 | 0.896 | | 0.649 | | | 1.309 | 0.350 | 19 | 0.898 | 0.716 | | | 1.362 | | | | 0.283 | | | |
|  | 180-350 days | 19,868 | 2 | 1.235 | | 0.817 | | | 1.772 | 0.183 | 9 | 1.013 | 0.796 | | | 1.409 | | | | 0.205 | | | |
|  | 351-667 days | 19,975 | 2 | 1.198 | | 0.810 | | | 1.709 | 0.190 | 6 | 0.859 | 0.701 | | | 1.270 | | | | 0.288 | | | |
|  | ≥668 days | 19,943 | 1 | 0.598 | | 0.327 | | | 0.827 | < 0.001 | 4 | 0.772 | 0.550 | | | 1.246 | | | | 0.461 | | | |
| **Paraproteinemia** | <180 days | 239,144 | 203 | Reference | |  | | |  |  | 222 | Reference |  | | | |  | | |  | | | |
|  | ≥180 days | 59,786 | 48 | 1.030 | | 0.891 | | | 1.364 | 0.104 | 86 | 1.058 | 0.847 | | | | 1.319 | | | 0.156 | | | |
|  | 180-350 days | 19,868 | 24 | 1.637 | | 1.013 | | | 2.055 | 0.043 | 39 | 1.582 | 0.984 | | | | 1.990 | | | 0.059 | | | |
|  | 351-667 days | 19,975 | 14 | 0.998 | | 0.790 | | | 1.217 | 0.201 | 28 | 0.948 | 0.764 | | | | 1.181 | | | 0.235 | | | |
|  | ≥668 days | 19,943 | 10 | 0.652 | | 0.531 | | | 0.820 | < 0.001 | 19 | 0.632 | 0.512 | | | | 0.790 | | | < 0.001 | | | |
| **Other polycythemia** | <180 days | 239,144 | 1,722 | Reference | |  | | |  |  | 2,017 | Reference |  | | | | |  | | | |  | |
|  | ≥180 days | 59,786 | 303 | 0.823 | | 0.668 | | | 0.934 | 0.017 | 598 | 0.790 | 0.642 | | | | | 0.989 | | | | 0.045 | |
|  | 180-350 days | 19,868 | 103 | 0.837 | | 0.682 | | | 0.972 | 0.036 | 246 | 0.803 | 0.653 | | | | | 1.012 | | | | 0.081 | |
|  | 351-667 days | 19,975 | 101 | 0.818 | | 0.663 | | | 0.897 | < 0.001 | 198 | 0.785 | 0.637 | | | | | 0.984 | | | | 0.042 | |
|  | ≥668 days | 19,943 | 99 | 0.809 | | 0.658 | | | 0.866 | < 0.001 | 154 | 0.778 | 0.628 | | | | | 0.957 | | | | 0.028 | |
| Abbreviations: sHR = subdistribution hazard ratio; CI, confidence interval; MDS, myelodysplastic syndrome  The subgroup of monocytic leukemia is not listed due to the lack of events in the first-event model.  †Competing variable was all-cause mortality. | | | | | | | | | | | | | | | | | | | | | | | |

| **Table O. Unadjusted (Crude) Hazard Ratios for Hematologic Neoplasm Risk Associated with Hypertension** | | | | | | | | | | | | |
| --- | --- | --- | --- | --- | --- | --- | --- | --- | --- | --- | --- | --- |
| **Cohorts** | **No Competing Risk Model** | | | | | | **Fine and Gray's Competing Risk Model**† | | | | | |
|  | **Crude HR ‡** | | **95% CI** | | ***P*** | | **Crude sHR**§ | | **95% CI** | | ***P*** | |
| Patient without Hypertension | Reference |  | |  | |  | Reference |  | |  | |  |
| Patients with Hypertension | 1.551 | 1.436 | | 1.734 | | < 0.001 | 1.521 | 1.418 | | 1.715 | | < 0.001 |
| Hydralazine < 180 days | 2.036 | 1.884 | | 2.113 | | < 0.001 | 1.974 | 1.857 | | 2.075 | | < 0.001 |
| Hydralazine ≥180 days | 1.183 | 1.096 | | 1.261 | | 0.002 | 1.148 | 1.080 | | 1.231 | | 0.010 |
| Abbreviations: HR = hazard ratio; sHR = subdistribution hazard ratio; CI = confidence interval  Patients without Hypertension: Patients with Hypertension = 4:1, 4-fold propensity score matching by age, sex, and season of index date  † Competing variable was all-cause mortality. | | | | | | | | | | | | |

| **Table P. Unadjusted (Crude) Subdistribution Hazard Ratios for Leave-One-Out Sensitivity Analysis**† | | | | | | | | | | |
| --- | --- | --- | --- | --- | --- | --- | --- | --- | --- | --- |
| **Subgroups of Hematologic Neoplasms** | **Prescription Duration of Hydralazine** | **First-Event Model** | | | | **Multiple-Event Model** | | | |  |
|  |  | **Crude HR**§ | **95% CI** | **95% CI** | ***P*** | **Crude sHR**§ | **95% CI** | **95% CI** | ***P*** |  |
| **Overall (Hematologic neoplasms)** | <180 days | Reference |  |  |  | - | - | - | - |  |
|  | ≥180 days | 0.770 | 0.645 | 0.927 | 0.014 | - | - | - | - |  |
|  | 180-350 days | 0.893 | 0.735 | 1.134 | 0.267 | - | - | - | - |  |
|  | 351-667 days | 0.735 | 0.592 | 0.934 | 0.017 | - | - | - | - |  |
|  | ≥668 days | 0.652 | 0.525 | 0.830 | < 0.001 | - | - | - | - |  |
| **Lymphosarcoma and reticulosarcoma** | <180 days | Reference |  |  |  | Reference |  |  |  |  |
|  | ≥180 days | 1.514 | 0.971 | 1.922 | 0.076 | 1.477 | 0.987 | 1.854 | 0.062 |  |
|  | 180-350 days | 2.281 | 1.040 | 2.897 | 0.030 | 2.228 | 1.027 | 2.794 | 0.037 |  |
|  | 351-667 days | 1.129 | 0.907 | 1.434 | 0.103 | 1.098 | 0.895 | 1.375 | 0.113 |  |
|  | ≥668 days | 0.902 | 0.525 | 1.286 | 0.472 | 1.110 | 0.900 | 1.382 | 0.101 |  |
| **Hodgkin's disease** | <180 days | Reference |  |  |  | Reference |  |  |  |  |
|  | ≥180 days | 1.281 | 0.967 | 1.627 | 0.074 | 1.242 | 0.956 | 1.569 | 0.071 |  |
|  | 180-350 days | 2.106 | 1.099 | 2.673 | 0.001 | 2.054 | 1.048 | 2.568 | 0.026 |  |
|  | 351-667 days | 0.999 | 0.837 | 1.323 | 0.163 | 1.018 | 0.812 | 1.234 | 0.197 |  |
|  | ≥668 days | 0.700 | 0.562 | 0.889 | < 0.001 | 0.679 | 0.535 | 0.840 | < 0.001 |  |
| **Other malignant neoplasms of lymphoid and histiocytic tissue** | <180 days | Reference |  |  |  | Reference |  |  |  |  |
|  | ≥180 days | 0.564 | 0.454 | 0.716 | < 0.001 | 0.551 | 0.444 | 0.694 | < 0.001 |  |
|  | 180-350 days | 0.729 | 0.586 | 0.926 | 0.013 | 0.712 | 0.573 | 0.893 | < 0.001 |  |
|  | 351-667 days | 0.521 | 0.419 | 0.663 | < 0.001 | 0.507 | 0.412 | 0.635 | < 0.001 |  |
|  | ≥668 days | 0.444 | 0.357 | 0.563 | < 0.001 | 0.432 | 0.347 | 0.552 | < 0.001 |  |
| **Multiple myeloma and immunoproliferative neoplasms** | <180 days | Reference |  |  |  | Reference |  |  |  |  |
|  | ≥180 days | 0.604 | 0.486 | 0.767 | < 0.001 | 0.605 | 0.493 | 0.755 | < 0.001 |  |
|  | 180-350 days | 0.663 | 0.532 | 0.841 | < 0.001 | 0.663 | 0.541 | 0.835 | < 0.001 |  |
|  | 351-667 days | 0.601 | 0.483 | 0.763 | < 0.001 | 0.602 | 0.488 | 0.749 | < 0.001 |  |
|  | ≥668 days | 0.550 | 0.441 | 0.698 | < 0.001 | 0.550 | 0.450 | 0.690 | < 0.001 |  |
| **Lymphoid leukemia** | <180 days | Reference |  |  |  | Reference |  |  |  |  |
|  | ≥180 days | 0.885 | 0.592 | 1.018 | 0.404 | 0.890 | 0.602 | 1.012 | 0.395 |  |
|  | 180-350 days | 1.005 | 0.889 | 1.404 | 0.111 | 1.111 | 0.900 | 1.394 | 0.103 |  |
|  | 351-667 days | 0.548 | 0.439 | 0.696 | < 0.001 | 0.550 | 0.453 | 0.699 | < 0.001 |  |
|  | ≥668 days | 0.413 | 0.332 | 0.525 | < 0.001 | 0.416 | 0.341 | 0.523 | < 0.001 |  |
| **Myeloid leukemia** | <180 days | Reference |  |  |  | Reference |  |  |  |  |
|  | ≥180 days | 0.706 | 0.567 | 0.897 | < 0.001 | 0.680 | 0.541 | 0.852 | < 0.001 |  |
|  | 180-350 days | 0.833 | 0.669 | 1.057 | 0.312 | 0.814 | 0.650 | 1.010 | 0.350 |  |
|  | 351-667 days | 0.732 | 0.590 | 0.930 | 0.015 | 0.712 | 0.568 | 0.882 | < 0.001 |  |
|  | ≥668 days | 0.553 | 0.444 | 0.702 | < 0.001 | 0.538 | 0.424 | 0.670 | < 0.001 |  |
| **Monocytic leukemia** | <180 days | Reference |  |  |  | Reference |  |  |  |  |
|  | ≥180 days | - | - | - | - | 0.908 | 0.577 | 2.021 | 0.433 |  |
|  | 180-350 days | - | - | - | - | 0.957 | 0.738 | 2.058 | 0.267 |  |
|  | 351-667 days | - | - | - | - | 0.694 | 0.423 | 1.418 | 0.571 |  |
|  | ≥668 days | - | - | - | - | 0.436 | 0.134 | 2.964 | 0.862 |  |
| **Other specified leukemia** | <180 days | Reference |  |  |  | Reference |  |  |  |  |
|  | ≥180 days | 0.909 | 0.731 | 1.154 | 0.268 | 0.887 | 0.721 | 1.114 | 0.279 |  |
|  | 180-350 days | 1.368 | 0.917 | 1.738 | 0.084 | 1.337 | 0.978 | 1.679 | 0.071 |  |
|  | 351-667 days | 1.036 | 0.874 | 1.401 | 0.125 | 1.323 | 0.862 | 1.662 | 0.133 |  |
|  | ≥668 days | 0.000 | - | - | 0.999 | 0.680 | 0.244 | 0.863 | < 0.001 |  |
| **Leukemia of unspecified cell type** | <180 days | Reference |  |  |  | Reference |  |  |  |  |
|  | ≥180 days | 0.766 | 0.616 | 0.974 | 0.037 | 0.766 | 0.625 | 0.965 | 0.032 |  |
|  | 180-350 days | 0.810 | 0.651 | 1.028 | 0.114 | 0.810 | 0.659 | 1.041 | 0.170 |  |
|  | 351-667 days | 0.762 | 0.612 | 0.967 | 0.034 | 0.762 | 0.620 | 0.953 | 0.027 |  |
|  | ≥668 days | 0.726 | 0.584 | 0.923 | 0.012 | 0.727 | 0.593 | 0.918 | 0.009 |  |
| **Neoplasm of uncertain behavior** | <180 days | Reference |  |  |  | Reference |  |  |  |  |
|  | ≥180 days | 0.729 | 0.586 | 0.927 | 0.013 | 0.714 | 0.579 | 0.894 | < 0.001 |  |
|  | 180-350 days | 0.966 | 0.776 | 1.227 | 0.235 | 0.944 | 0.764 | 1.169 | 0.239 |  |
|  | 351-667 days | 0.692 | 0.557 | 0.879 | < 0.001 | 0.676 | 0.549 | 0.849 | < 0.001 |  |
|  | ≥668 days | 0.531 | 0.427 | 0.675 | < 0.001 | 0.523 | 0.421 | 0.654 | < 0.001 |  |
| **MDS** | <180 days | Reference |  |  |  | Reference |  |  |  |  |
|  | ≥180 days | 0.870 | 0.625 | 1.287 | 0.374 | 0.875 | 0.698 | 1.327 | 0.304 |  |
|  | 180-350 days | 1.201 | 0.784 | 1.742 | 0.219 | 0.987 | 0.775 | 1.373 | 0.226 |  |
|  | 351-667 days | 1.166 | 0.780 | 1.679 | 0.223 | 0.836 | 0.683 | 1.237 | 0.317 |  |
|  | ≥668 days | 0.582 | 0.317 | 0.812 | < 0.001 | 0.752 | 0.536 | 1.214 | 0.465 |  |
| **Paraproteinemia** | <180 days | Reference |  |  |  | Reference |  |  |  |  |
|  | ≥180 days | 0.999 | 0.849 | 1.341 | 0.158 | 1.031 | 0.825 | 1.285 | 0.172 |  |
|  | 180-350 days | 1.592 | 0.980 | 2.021 | 0.083 | 1.541 | 0.959 | 1.938 | 0.066 |  |
|  | 351-667 days | 0.945 | 0.760 | 1.201 | 0.240 | 0.923 | 0.744 | 1.150 | 0.258 |  |
|  | ≥668 days | 0.635 | 0.510 | 0.806 | < 0.001 | 0.615 | 0.499 | 0.769 | < 0.001 |  |
| **Familial polycythemia** | <180 days | Reference |  |  |  | Reference |  |  |  |  |
|  | ≥180 days | 0.915 | 0.438 | 1.255 | 0.566 | 0.894 | 0.459 | 1.230 | 0.542 |  |
|  | 180-350 days | 0.752 | 0.415 | 1.195 | 0.584 | 0.649 | 0.346 | 1.073 | 0.657 |  |
|  | 351-667 days | - | - | - | - | 0.914 | 0.486 | 1.247 | 0.519 |  |
|  | ≥668 days | 1.000 | 0.597 | 1.449 | 0.402 | 0.997 | 0.509 | 1.336 | 0.401 |  |
| Abbreviations: PYs = Person-years; HR = Hazard Ratio; sHR = subdistribution hazard ratio; CI = confidence interval; MDS, myelodysplastic syndrome.  †Competing variable was all-cause mortality. | | | | | | | | | | |

| **Table Q. Unadjusted (Crude) Hazard Ratios for Mortality Analysis** | | | | | | | | | | | | | | | | | | | | | |
| --- | --- | --- | --- | --- | --- | --- | --- | --- | --- | --- | --- | --- | --- | --- | --- | --- | --- | --- | --- | --- | --- |
| **Prescription Duration of Hydralazine** | | **≥180 days** | | | | | **<180 days** | | | | | | | | **≥180 days vs. <180 days**  (Reference) | | | | | | |
| **Mortality Analysis** | | **Events** | **PYs** | **Rate (per 10^5^ PYs)** | | | | **Events** | | **PYs** | **Rate (per 10^5^ PYs)** | | | | **Crude HR** | **95% CI** | | ***P*** | | |  |
| **Hematologic neoplasm-related mortality** | 153 | | 768,980.24 | | 19.90 | 661 | | | 2,897,605.18 | | | 22.81 | | 0.907 | | 0.641 | 1.284 | 0.358 | | |  |
| Lymphosarcoma and reticulosarcoma | 3 | | 768,980.24 | | 0.39 | 11 | | | 2,897,605.18 | | | 0.38 | | 1.069 | | 0.755 | 1.513 | 0.247 | | |  |
| Hodgkin's disease | 7 | | 768,980.24 | | 0.91 | 28 | | | 2,897,605.18 | | | 0.97 | | 0.980 | | 0.692 | 1.386 | 0.303 | | |  |
| Other malignant neoplasms of lymphoid and histiocytic tissue | 20 | | 768,980.24 | | 2.60 | 103 | | | 2,897,605.18 | | | 3.55 | | 0.761 | | 0.538 | 1.077 | 0.467 | | |  |
| Multiple myeloma and immunoproliferative neoplasms | 15 | | 768,980.24 | | 1.95 | 79 | | | 2,897,605.18 | | | 2.73 | | 0.744 | | 0.526 | 1.053 | 0.091 | | |  |
| Lymphoid leukemia | 9 | | 768,980.24 | | 1.17 | 48 | | | 2,897,605.18 | | | 1.66 | | 0.734 | | 0.520 | 1.040 | 0.082 | | |  |
| Myeloid leukemia | 11 | | 768,980.24 | | 1.43 | 56 | | | 2,897,605.18 | | | 1.93 | | 0.770 | | 0.544 | 1.090 | 0.460 | | |  |
| Monocytic leukemia | 0 | | 768,980.24 | | 0.00 | 0 | | | 2,897,605.18 | | | 0.00 | | - | | - | - | - | | |  |
| Other specified leukemia | 0 | | 768,980.24 | | 0.00 | 2 | | | 2,897,605.18 | | | 0.07 | | 0.000 | | - | - | 0.999 | | |  |
| Leukemia of unspecified cell type | 21 | | 768,980.24 | | 2.73 | 72 | | | 2,897,605.18 | | | 2.48 | | 1.143 | | 0.808 | 1.618 | 0.194 | | |  |
| Neoplasm of uncertain behavior | 10 | | 768,980.24 | | 1.30 | 53 | | | 2,897,605.18 | | | 1.83 | | 0.740 | | 0.523 | 1.046 | 0.075 | | |  |
| MDS | 2 | | 768,980.24 | | 0.26 | 7 | | | 2,897,605.18 | | | 0.24 | 1.120 | | | 0.791 | 1.584 | | 0.206 |  |  |
| Paraproteinemia | 25 | | 768,980.24 | | 3.25 | 96 | | | 2,897,605.18 | | | 3.31 | 1.021 | | | 0.721 | 1.444 | | 0.279 |  |  |
| Other polycythemia | 30 | | 768,980.24 | | 3.90 | 106 | | | 2,897,605.18 | | | 3.66 | 1.109 | | | 0.783 | 1.570 | | 0.218 |  |  |
| **All-cause mortality** | 6,789 | | 768,980.24 | | 882.86 | 24,121 | | | 2,897,605.18 | | | 832.45 | 1.103 | | | 0.779 | 1.562 | | 0.225 |  |  |
| Abbreviations: PYs, person-years; CI, confidence interval; MDS, myelodysplastic syndrome; HR, hazard ratio | | | | | | | | | | | | | | | | | | | | | |
