# Supplementary material for: Association of hydralazine use with risk of hematologic neoplasms in patients with hypertension: A nationwide population-based cohort study in Taiwan
Source: PLoS Med. 2025 Dec 4;22(12):e1004646. doi: 10.1371/journal.pmed.1004646 (PMC12677456; doi:10.1371/journal.pmed.1004646)
Supplement: Attachment — Submitted filename: Comments.docx [file pmed.1004646.s003.docx]

Dear Editor,

I would like to thank you for the opportunity that you gave me to review the manuscript “Hydralazine Reduces the Risk of Hematologic Neoplasms in Patients with Hypertension: A Nationwide Population-Based Cohort Study in Taiwan” (PMEDICINE-D-25-01670R1). While the research approach and the findings are quite interesting and well-organized, some issues should be addressed to make it worthy of publication. Please find below my comments and suggestions regarding your work.

Comments

Abstract

-Please also provide the p-values for each comparison that you describe.

Introduction

-Indeed, hypertension constitutes a significant clinical issue in patients with hematological malignancies, especially for allogeneic hematopoietic cell transplantation recipients. Endothelial injury has a critical role in the pathogenesis of hypertension in this setting, and this should be highlighted in the introduction section (<https://doi.org/10.1016/j.tru.2024.100186>). Moreover, novel agents, such as some TKIs, might lead to the development of drug-induced hypertension (<https://doi.org/10.1016/j.blre.2019.03.003>).

Methods

-In line 142, I would change “eg” with such as.

-Some more data regarding the definition of health insurance premiums and NTD are essential.

-“Statistical significance was set at a 2-tailed P value of < .001.” Why did you use such a p-value and not p<0.05?

Results

-In lines 296-298, please include the exact p-values.

Discussion

-
