# Supplementary material for: Association of hydralazine use with risk of hematologic neoplasms in patients with hypertension: A nationwide population-based cohort study in Taiwan
Source: PLoS Med. 2025 Dec 4;22(12):e1004646. doi: 10.1371/journal.pmed.1004646 (PMC12677456; doi:10.1371/journal.pmed.1004646)
Supplement: Attachment — Submitted filename: renamed_21205.docx [file pmed.1004646.s005.docx]

### Point-by-Point Response to Editors' Comments

**GENERAL**

- **Comment 1:** Please confirm that your title complies with to PLOS Medicine's style. Your title must be nondeclarative and not a question. It should begin with main concept if possible. "Effect of" should be used only if causality can be inferred, i.e., for an RCT. Please place the study design ("A randomized controlled trial," "A retrospective study," "A modelling study," etc.) in the subtitle (i.e., after a colon).
- **Response:**

Thank you for this guidance. We have revised the title to be non-declarative and have added the study design as a subtitle.

- **Change Made:** The **title** has been changed to: "**Association of hydralazine use with risk of hematologic neoplasms in patients with hypertension: a nationwide population-based cohort study in Taiwan**". (**page 1, lines 1–3**)
  We also revised the **short title** in submission system to “**Hydralazine use and hematologic neoplasm risk in patients with hypertension**”.
- **Comment 2:**
  1. Statistical reporting: Please revise throughout the manuscript, including tables and Figures.

- Please report statistical information as follows to improve clarity for the reader ""XX

(95% CI [XX,XX]; p</=)"".
 - Please separate upper and lower bounds with commas instead of hyphens as the

latter can be confused with reporting of negative values.

1. Please repeat statistical definitions (HR, CI etc.) for each set of parentheses.
   - **Response:**
2. Thank you for the detailed and constructive feedback on our statistical reporting. We agree that consistency and clarity are paramount for presenting complex data and appreciate the specific guidance provided. **We have fully adopted the requested format—XX (95% CI [XX,XX]; *P*-value)—using commas to separate confidence interval bounds throughout the abstract and main text of the manuscript**.

As the tabular format already presents the point estimates (e.g., aHR, adjusted sHR) and their corresponding confidence intervals in clearly delineated, separate columns, we believe this structure provides maximum clarity. Therefore, **we did not replicate the full parenthetical format within the table cells**, as it would be redundant. We are confident that this approach fulfills the goal of clear, unambiguous reporting while maintaining a well-organized and readable presentation for all components of the manuscript.

1. Regarding the suggestion to repeat statistical definitions (such as HR and CI) for each set of parentheses, we have carefully considered its implementation across the manuscript. While we understand the goal is to make the data as clear as possible, **we are slightly concerned that repeating these definitions in every instance**, **especially in paragraphs dense with results, may inadvertently make the text cumbersome and hinder overall readability**.

Therefore, **we would like to respectfully propose a compromise**. Our suggestion is to define the terms **upon *their first use for each paragraph*,** rather than **repeating the definitions in every set of parentheses.** We believe this approach effectively balances clarity and conciseness. It ensures the reader has the necessary definitions readily available in the immediate context without having to look far, while preventing the text from becoming overly repetitive.

- **Change in Manuscript:**

1. All statistical values are now presented in the format "XX (95% CI [XX, XX]; *P* < .XXX)", including the following revisions: **page 3, line 50; page 4, lines 54–57; page 14, lines 314–315; page 15, line 322; page 15, lines 328–329; page 15, lines 338–341; page 17, line 376; pages 17–18, lines 390–397; page 18, lines 401–402; page 18, lines 403–404; page 21, lines 488–489; page 22, lines 508–509.**
2. As per our compromise proposal, we repeat statistical definitions (aHR, adjusted sHR, and CI) for each paragraph **(page 3, lines 49–50; page 12, line 247; page 12, line 261; page 13, lines 274–275; page 14, lines 314–315; page 15, lines 338–339; page 17, line 374; page 17, line 376; page 17, line 390–391; page 18, line 401; page 21, lines 488–489; page 22, lines 508–509).**

Furthermore, we have added a specific sentence to the **Statistical Analysis** section to explicitly and clearly define the key statistical terms used in our primary analyses upfront: The hematologic neoplasm risk was estimated using two models: adjusted hazard ratios (adjusted HRs, based on Cox regression), and adjusted subdistribution hazard ratios (adjusted sHRs, based on Fine and Gray’s competing risk models) (**Table 2**) **(page 13, lines 273–276).**

- **Comment 3:** Please ensure that all abbreviations are defined at first use throughout the text (including statistical abbreviations). Please also check figures and tables.
- **Response:**

We have carefully reviewed the manuscript, tables, and figures to ensure every abbreviation is defined upon its first use in each section (main text, abstract, tables, figures).

- **Comment 4:** Please ensure that tables and figures, including those in supplementary files, are appropriately referenced in the main text.
- **Response:**

We have cross-checked the manuscript to confirm that all tables and figures, including supplementary materials, are correctly and sequentially referenced in the main text.

- **Comment 5:** Please check that any use of statistical terms (such as trend or significant) are supported by the data, and if not please remove them. Please note that the term trend should be used only when the test for trend has been conducted.
- **Response:**

Thank you for this important reminder. We have reviewed the manuscript and removed the term "trend" where a formal test for trend was not performed. All uses of the word "significant" are now directly supported by *P*-values that meet the defined statistical threshold for the respective analysis.

- **Change in Manuscript:** We have meticulously reviewed the entire manuscript to ensure that all statistical terms are precisely supported by the data, in accordance with the editor's guidance.
  1. **Removal of the term "trend"**: We have replaced "trend" with more appropriate, descriptive language such as "duration-dependent association" **(page 15, lines 334–335),** or "duration-dependent pattern" **(page 16, line 348),** or "pattern" **(page 17, lines 379–380)** to describe the observed data without making an unsupported statistical claim.
  2. **Verification of the term "significant"**: We conducted a thorough check of every use of the word "significant." We have revised several key passages (listed below) to ensure this term is used only when the corresponding *P*-value meets our pre-specified threshold of ***P* < .001** for all analyses.
     - **Baseline Characteristics (page 14, lines 304–307):** The text describing baseline differences **in Table 1** was revised to only label findings as "significant" if they met the *P* < .001 threshold (e.g., for congestive heart failure, ischemic heart disease, etc.). We have removed the incorrect claims of significance for variables like proteinuria (*P* = .044) and asthma (*P* = .002) that were present in the previous manuscript.
     - **Risk Factors (page 15, lines 320–323 & lines 326–330):** The text describing other risk factors **in Table 2** was revised to explicitly state that the association for mebendazole (***P* = .003**) "did not meet our pre-specified significance threshold." The text also now clarifies that for aspirin (***P* = .014**), the association did not meet the threshold, whereas those for celecoxib and thalidomide (**both *P* < .001**) did.
     - **Sensitivity Analysis (pages 17–18, lines 388–397):** The text describing the sensitivity analysis in **Table 4** was corrected. We now accurately state that the association for the overall ≥ 180 days group did not meet our threshold in the 5-year exclusion analysis (*P* = .001), while clarifying that the association for the longest-duration subgroup (≥ 668 days) *did* remain statistically significant (***P* < .001**).
- **Comment 6:** Please review your text for claims of novelty or primacy (e.g. 'for the first time') and remove this language.
- **Response:**

We have removed all claims of novelty, such as "the first," from the manuscript to present our findings more objectively.

- **Change in Manuscript:** The sentence in the **Discussion** now reads: " This nationwide retrospective cohort study investigated the association between the use of AHAs and the risk of developing hematologic neoplasms in patients with hypertension." **(page 18, lines 411–413)**, in accordance with all editor’s comments.
- **Comment 7:** Your study is observational and therefore causality cannot be inferred. Please remove language that implies causality, such as effect. Refer to associations instead. Please revise throughout.
- **Response:**

Thank you for this essential reminder. We fully agree that our observational study design does not allow for causal inferences and that our previous draft contained too strong language. We have performed a comprehensive, word-by-word review of the entire manuscript to remove all language that implies causality.

We have systematically replaced causal terms (e.g., "reduces," "effect," "inhibit") with appropriate associative language (**e.g., "association with," "linked to"**).

- **Change in Manuscript:** this systematic revision has been applied throughout the entire manuscript. Key changes include:
  1. **Main Title:** The title has been changed from its original declarative form ("Hydralazine Reduces the Risk...") to the associative "**Association of hydralazine use with risk of hematologic neoplasms**...". (**page 1, lines 1–3**)
  2. **Abstract section:** The phrase "the potential chemopreventive effects of continuous hydralazine (HLZ) use, an antihypertensive agent (AHA) with notable anti-HN activity, compared to other AHAs, remain uncertain." has been revised to " **it remains unknown whether continuous use of hydralazine—an antihypertensive agent (AHA) with notable anti-neoplastic activity—is associated with a lower risk of hematologic neoplasms compared to other AHAs**." **(page 3, lines 31–34)**
  3. **Introduction:**
- "reported to attenuate the development of" was revised to "**been associated with a lower risk of**".**(page 6, lines 104–105)**
- "AHA-based chemoprevention of" was revised to "**investigation for its potential association with the risk of**".**(page 7, lines 133–134)**
  1. **Results:**
     - The phrase "preventive effect on HN risk" has been changed to "**was associated with a lower risk of hematologic neoplasms**" **(page 15, line 321).**
     - The phrase " exerted a protective effect against HNs" has been changed to "**were linked to a lower risk**" **(page 15, line 325).**
     - The phase "HLZ demonstrated superior potential in inhibiting the subsequent development of leukemia of unspecified cell type" has been changed to "**HLZ was associated with a lower risk of subsequent development of leukemia of unspecified cell type**" **(page 16, lines 352–353).**
  2. **Figure/Section Titles**:
- The title of **figure 2**, "Long-Term Effect of Hydralazine on the Incidence of Hematologic Neoplasms in Patients with Hypertension, 2000-2015." was changed to "**Association of Long-Term Hydralazine Use with the Incidence of Hematologic Neoplasms in Patients with Hypertension, 2000-2015.**"
- The "Long-term effect of..." heading was changed to "**Association of long-term hydralazine use with**..." **(page 16, line 367).**
  1. **Discussion and Conclusion:**
     - The phrase, "HLZ may exert favorable effects through several biological regulatory mechanisms" was changed to "**this finding suggests the association may be linked to several biological regulatory mechanisms**" **(page 19, lines 434–436)**.
     - The phrase, "confirming HLZ as a valid protective agent" was rephrased to "**supporting the robustness of the observed association for overall hematologic neoplasms**" **(page 24, lines 554–555)** to avoid causal inference.
     - The phrase, "HLZ could be considered for drug repurposing in HN prevention" was rephrased to "our results highlight hydralazine as a compelling candidate for drug repurposing to address the risk of hematologic neoplasms" **(page 25, lines 571–572).**
     - The phase, "HLZ may mitigate the risk of HN" was changed to "**hydralazine use may be associated with a lower risk of hematologic neoplasm**" **(page 25, lines 556–558).**
     - The phase, "prospective studies are warranted to confirm this effect" was changed to "**prospective studies are warranted to further investigate this association and its potential clinical implications**" **(page 25, lines 572–574).**
     - The final Conclusion was revised from "may offer chemopreventive effects" to "**may be linked to a lower incidence**" **(page 25, lines 578–579).**
- **Comment 8:** Please confirm that all statements (data availability, funding etc.) in the online submission form are accurate.
- **Response:**

Thank you for the reminder. We have carefully reviewed all metadata and statements entered into the online submission form.

We confirm that all information—including the **Data Availability Statement, the Funding statement, author contributions, and potential conflicts of interest**—is accurate, complete, and fully consistent with the contents of the revised manuscript.

- **Comment 9:** Please include the statement on code availability in the data availability statement.
- **Response:**

We have moved the code availability statement into the data availability section as requested.

- **Change in Manuscript:** The "Data Availability Statement" now includes the sentence: "**Statistical analysis was performed using SPSS and STATA software. No custom code was generated for the analysis**." **(page 27, lines 621–622)**
- **Comment 10:** Please reduce the number of abbreviations throughout the manuscript to improve readability. We suggest to spell out exposure group, reference group, hypertension, hematologic neoplasms, and hydralazine.
- **Response:**

Thank you for this valuable suggestion to improve the manuscript's readability. We agree that reducing the number of abbreviations for the study's core concepts makes the text clearer and more accessible to a broad audience. We have performed a comprehensive revision of the entire manuscript to spell out the five key terms as requested. In accordance with your request, we have **removed the abbreviations** **for the five key terms, "EG, exposure group"; "RG, reference group"; "HTN, hypertension"; "HNs, hematologic neoplasms" and "HLZ, hydralazine"** you highlighted.

- **Comment 11:** Please revise for use of patient-centered language. Please note that patient-centered language is constructed with the use of post-modified nouns (e.g. 'patients with hypertension’ (or similar) instead of ‘hypertensive patients’) putting the person first in the sentence structure.
- **Response:**

Thank you for this important guidance. We fully agree with the principle of using patient-centered language and putting the person first. We have already implemented this change **throughout the manuscript, figures and tables**, consistently using phrases such as **"patients with hypertension"** and **"patients without hypertension"**.

- **Comment 12:** The terms gender and sex are not interchangeable... please use the appropriate term and revise throughout (including figures and tables).
- **Response:**

Thank you for this important clarification. We agree that the distinction between "sex" and "gender" is a crucial one. Our data is sourced from Taiwan's National Health Insurance Research Database, which records the biological sex of patients, not their gender identity.

Therefore, "sex" is the correct term for our study. We have carefully reviewed the entire manuscript to ensure this is used consistently. **We have corrected the terminology from "gender" to "sex" throughout the manuscript and tables**.

**ABSTRACT**

- **Comment 1:** Please confirm that your abstract complies with our requirements...
- **Response:**

We have revised the abstract to ensure it meets all structural and content requirements for a cohort study as per the journal's guidelines.

- **Comment 2:** Please provide the main baseline characteristics of the study population.
- **Response:**

Thank you for this suggestion. We agree that including the main baseline characteristics of the study population in the abstract is essential for providing context to the reader.

- **Change in Manuscript:** The abstract now includes the following: "**Both groups were well-matched, with a mean age of approximately 60.8 years and 52.19% male**." **(page 3, lines 42–43)**
- **Comment 3:** In the last sentence of the Abstract Methods and Findings section, please describe the main limitation(s) of the study's methodology.
- **Response:**

We have added the main study limitation to the end of the Methods and Findings section of the abstract.

- **Change in Manuscript:** The last sentence now reads: "**The main limitation was the potential for residual confounding due to the unavailability of lifestyle and laboratory data in the administrative database.**" **(page 4, lines 57–59)**
- **Comment 4:** Please confirm that all numbers presented in the abstract are present and identical to numbers presented in the main manuscript text.
- **Response:**

We have verified that all numerical data in the abstract are identical to those presented in the main text and tables.

- **Comment 5:** Please reduce the number of abbreviations used in the abstract.
- **Response:**

Thank you for this feedback. We agree that the previous abstract was overly dense with abbreviations, which hindered its readability for a general audience. We have thoroughly revised the abstract to address this issue by prioritizing clarity and spelling out all key terms, such as "exposure group," "reference group," "hydralazine," "hematologic neoplasms," and "hypertension."

The only abbreviations that remain in the **Abstract** are "**AHA" (antihypertensive agent)** and "**adjusted sHR" (adjusted subdistribution hazard ratio)**, both of which are standard, defined at first use, and are necessary for conciseness.

- **Comment 6:** “adjusted risk difference, RD: 9 [95%CI:2, 16] per 10,000.” – please note that in the following brackets, you have reported the risk difference with a percentage sign.
- **Response:**

We thank the editor for noting this. The **Abstract** provided in our manuscript **does not report an adjusted risk difference calculation**. Our primary analyses and all reported results focus on adjusted hazard ratios (HR) and adjusted subdistribution hazard ratios (sHR) from Cox regression and competing risk models.

We have reviewed the revised **Abstract** to ensure all statistical reporting is accurate and in the correct format.

- **Comment 7:** “Subgroup analyses revealed lower adjusted sHRs with longer HLZ prescription durations..” – Please specify 'longer duration'.
- **Response:**

Thank you for this valuable feedback. You are correct that the original phrasing in the abstract was vague and did not adequately specify which "longer duration" the statistical examples referred to. The values we had cited (e.g., adjusted sHR 0.707 for myeloid leukemia) were for the **overall** **≥ 180 days** category, which did not clearly illustrate the duration-dependent nature of the association. To provide the requested specificity and to more accurately highlight our key findings, we have revised this sentence to use the **longest duration category (≥ 668 days)** as the prime example, as this best demonstrates the association.

- **Change in Manuscript:** The sentence has been revised to: "Subgroup analyses demonstrated that the association with a lower risk was strongest in the longest prescription duration category. For example, for patients with prescription durations of ≥668 days, the adjusted sHR was 0.448 (95% CI [0.366,0.555]; *P* < .001) for other malignant neoplasms of lymphoid and histiocytic tissue, 0.552 (95% CI [0.453,0.683]; *P* < .001) for multiple myeloma and immunoproliferative neoplasms, and 0.555 (95% CI [0.457,0.689]; *P* < .001) for myeloid leukemia." **(page 4, lines 51–57)**
- **Comment 8:** “Conversely, users of other anti-HTN medications showed higher adjusted sHRs for overall HN in this cohort.” – we suggest providing one example.
- **Response:**

Thank you for pointing this out. We agree this sentence was confusing and disrupted the logical flow of the **Abstract**.

The confusing sentence was referred from **Supplemental Table S3**, which compared the **reference group** (patients with hypertension taking hydralazine < 180 days but **other antihypertensive agents ≥180 days**) to a **different comparator** (**patients without hypertension**), showing that **reference group** a higher risk (adjusted sHR = 1.924) when compared to the **patients without hypertension**.

However, the main narrative of our **Abstract** is the direct comparison between the **exposure group** (hydralazine ≥180 days) and the **reference group** (hydralazine <180 days), as presented in **Table 2**.

To avoid misinterpretation, we have **removed this sentence** from the **Abstract** as it does not add to the core finding.

- **Comment 9:** “dose-dependently” – why dose-dependently?
- **Response:**

Thank you for this precise and important correction. You are absolutely correct. Our study analyzed the association based on the **duration** of prescription (e.g., ≥ 180 days, 351-667 days), not dosages (e.g., mg/day). The term "dose-dependently" was therefore inaccurate and misleading. We have removed the term "dose-dependently" from the **Abstract**'s conclusion and replaced it with "duration-dependent" to accurately reflect our methodology.

- **Change in Manuscript:** The **Abstract** now states: "In this study, we observed that long-term hydralazine use in patients with hypertension was associated with a lower, **duration-dependent** risk of hematologic neoplasms. " **(page 4, lines 61–62)**
- **Comment 10:** Abstract Conclusions: Please address the study implications without overreaching what can be concluded from the data; the phrase "In this study, we observed ..." may be useful. Please interpret the study based on the results presented in the abstract, emphasizing what is new without overstating your conclusions.
- **Response:**

We have revised the conclusion of the **Abstract** to be more circumspect and directly reflective of our observational findings, using the suggested phrasing.

- **Change in Manuscript:** The conclusion now states: "**In this study, we observed** that **long-term hydralazine use** in patients with hypertension was associated with a lower, **duration-dependent risk of hematologic neoplasms**. **These findings warrant prospective studies** to confirm this association and its **potential clinical implications**." **(page 4, lines 61–64)**

**AUTHOR SUMMARY**

- **Comment 1:** Please refer to our previous comment about reducing the number of abbreviations.
- **Response:**

Thank you for this reminder. We agree that the previous version of the Author Summary was not sufficiently accessible for a non-scientific audience and relied too heavily on technical abbreviations. We have **completely rewritten the Author Summary** to be a clear, streamlined, non-technical narrative, in full accordance with your feedback.

- **Comment 2:** Please streamline the Author Summary. We suggest focusing on the main analysis and removing references to subgroups. The Author Summary should be a nontechnical summary of your research, making your findings accessible to both scientists and non-scientists. For example, "adjusted subdistribution hazard ratio" is not lay language. Also, the Author Summary should be distinct from the scientific abstract.
- **Response:**

Thank you for this valuable advice. We have completely rewritten the **Author Summary** to be a clear, non-technical overview. We removed jargon, focused on the main findings, and ensured it is distinct from the **Abstract**.

We have revised the **Author Summary** to fully align these instructions. Specifically, we have:

1. **Streamlined and Simplified:** Rephrased the "Why was this study done?" section to clearly explain the research gap in plain language.
2. **Focused on Main Analysis:** Removed all references to specific subgroups from all sections.
3. **Removed Technical Jargon:** Replaced technical terms like "adjusted subdistribution hazard ratio" with the plain-language finding ("**approximately 21% lower risk**").
4. **Replaced specific medical terminology:** We have replaced "hypertension" with "**high blood pressure**" and replaced "hematologic neoplasms" with "**blood cancers**".
5. **Added Limitations:** We have also ensured that the "What do these findings mean?" section now includes a clear statement on **the study's main limitations, particularly the observational design and inability to prove causality**, as requested.

- **Change in Manuscript: the Author Summary has been completely rewritten (Pages 4–5, Lines 66–93).**

**METHODS AND RESULTS**

- **Comment 1:** You have stated that statistical significance was set at a 2-tailed P value of <.001. However, you reported significant differences for proteinuria (0.044) and asthma (0.002). Please clarify.
- **Response:**

Thank you for pointing out this critical inconsistency. We apologize for the lack of clarity in our previous manuscript. To maintain the highest level of statistical rigor and ensure consistency throughout the manuscript, we have applied the stringent *P* < .001 threshold to all statistical comparisons, including the baseline characteristics in Table 1.

You are therefore correct that the differences observed for **proteinuria (*P* = .044) and asthma (*P* = .002) do not meet our study's definition of statistical significance**. We have revised the "**Patient selection and characterization**" section accordingly to remove any claims of significance for these variables.

- **Change in Manuscript:** The revised paragraph now accurately reads:

"The exposure group had a significantly higher comorbidity burden, with higher rates of congestive heart failure (CHF), ischemic heart disease (IHD), malignant neoplasm of the kidney/renal pelvis, and acute glomerulonephritis/nephrotic syndrome, as well as a higher overall Charlson Comorbidity Index_revised (CCI_R) score." **(page 14, lines 304–307)**

- **Comment 2:** Table 1: We suggest listing "normal pregnancy" separately from "comorbidities." Also, please define "normal pregnancy”.
- **Response:**

Thank you for this sensible suggestion. We agree that "Normal pregnancy" is not a comorbidity and should be listed as a separate variable. We have updated our tables to reflect this distinction. Furthermore, as a consequence of this re-categorization, "Normal pregnancy" has also been treated as a separate covariate in our main multivariable risk analysis (**Table 2**).

- **Change in Manuscript:** We have made the following revisions throughout the manuscript and supplemental files:

1. **Definition**: In **S1 Table**, "Normal pregnancy" is now defined by its corresponding *ICD-9-CM* code, V22.
2. **Baseline Tables**: In all baseline characteristics tables (**Table 1, S0 Table, S8 Table, S10 Table**), "Normal pregnancy" has been moved from the "Comorbidities" list and is now presented as a separate main-level variable.
3. **Analysis Table:** Reflecting this change, "Normal pregnancy" is now also included as a distinct covariate in our multivariable risk regression analysis in **Table 2** and is no longer grouped under the "Comorbidities" sub-heading.

- **Comment 3:** Some of the results that were described as significant (e.g. mebendazole l.305, aspirin l.310) don’t seem to fall within the threshold for statistical significance that you set. Please comment on this and revise accordingly.
- **Response:**

Thank you for your sharp observation and for upholding the rigor of our statistical reporting. You are absolutely correct. In our previous draft, we had inappropriately used the term "significant" for findings related to mebendazole (*P* = .003) and aspirin (*P* = .014), even though their *P*-values did not meet our study's pre-specified significance threshold of *P* < .001 for outcome analyses. We apologize for this oversight. We have revised entire section to be precise, clearly stating which associations met our stringent threshold and which did not.

- **Change in Manuscript:** we have revised the text to explicitly state:
  1. “**Mebendazole**, which has recognized antileukemia activity, was associated with **a lower risk** of hematologic neoplasms (adjusted sHR = 0.696, 95% CI [0.286,0.957]; *P* = .003), although this association **did not meet our pre-specified significance threshold**.” **(page 15, lines 320–323)**
  2. The revised paragraph, “However, **a higher risk** of hematologic neoplasms was associated with the use of **aspirin**, celecoxib, statins, and thalidomide—with the associations for celecoxib (adjusted sHR = 1.608, 95% CI [1.163,1.933]; *P* < .001) and thalidomide (adjusted sHR = 1.846, 95% CI [1.194,2.466]; *P* < .001) **meeting our pre-specified significance threshold**” **(page 15, lines 326–330)** now clarifies that while a higher risk was observed for aspirin, **only the associations for celecoxib and thalidomide met our stringent significance threshold, thereby correcting the previous inaccuracy regarding aspirin**.
- **Comment 4:** ll.306-309, “Other medications, including itraconazole [44], valproate [45], and metformin [46], which have been reported to inhibit HN progression, exerted a protective effect against HNs, although significance was not reached.” – Based on the results presented in Table 2, we find this statement inaccurate. Please revise.
- **Response:**

Thank you for identifying this clear inaccuracy. Our previous statement was factually wrong, particularly regarding valproate, which our data (**Table 2**) showed was associated with a higher, not lower risk (adjusted sHR = 1.475). We have completely rewritten this sentence to accurately reflect the data as presented in **Table 2**.

- **Change in Manuscript:** The revised sentence now reads: "Similarly, **no statistically significant associations** were observed for other medications investigated: **itraconazole** and **metformin** were linked to a lower risk, whereas **valproate** was linked to a higher risk." **(page 15, lines 323–326)**
- **Comment 5:** l.323ff, “This trend was consistently observed across several HN subgroups...” – Please revise for accuracy.
- **Response:**

Thank you for this important note on accuracy. We agree that our previous phrasing, "consistently observed," was an overstatement. While the data in **previous** **Table 3** shows that the point estimates (the adjusted HRs) trend downward with longer duration for all the subgroups we listed, the association was not statistically significant at every single duration interval for all of them (e.g., **for lymphoid leukemia at 180-350 days,** ***P* = .189**).

To correct this overstatement and improve accuracy, we have removed the word "consistently" and rephrased the sentence by **highlighting the** **specific subgroups that show a significant association (*P* < .001) at all duration intervals**.

- **Change in Manuscript:** The sentence now reads: “A statistically significant, inverse duration-response association was observed between hydralazine use and hematologic neoplasm risk across several subgroups. This association was most pronounced for other malignant neoplasms of lymphoid and histiocytic tissue, multiple myeloma and immunoproliferative neoplasms, and other polycythemia.” **(pages 15–16, lines 342–346)**
- **Comment 6:** l.330, “Notably, HLZ demonstrated superior potential in inhibiting the subsequent development of leukemia of unspecified cell type, as shown in the multiple-event model.” – please provide a reference to the relevant table/graph.
- **Response:**
- Thank you for noting this omission. We agree that this claim requires a direct reference to the supporting data. We have revised the sentence to include a specific reference to **S2 Table**, which contains the results of the multiple-event model analysis.
- **Change in Manuscript:** The sentence now reads: "Notably, hydralazine **was associated with a lower risk of** subsequent development of leukemia of unspecified cell type, as shown in the multiple-event model **(S2 Table)**." **(page 16, lines 352–354)**
- **Comment 7:** We understand that the paragraph "Association of HN incidence stratified by HN subgroup and duration of HLZ prescription" as well as Table 3 contain a lot of information. We believe that the results could be presented more clearly and with better structure. Please revise accordingly. Consider focusing on a few HN subgroups in the main text and moving some of the results to the supplementary material.
- **Response:**

Thank you for this constructive feedback. We agree completely that the original presentation of the stratified subgroup analysis, in both the paragraph and the accompanying table, was dense and could be significantly improved for clarity and focus. To address this, we have substantially restructured this section. **We have streamlined the main text to focus on the most notable findings** and have split the original, large Table 3. The main manuscript now contains a more concise table **(the new Table 3)** that highlights the key results, while the full, detailed breakdown for all other subgroups has been moved to a new table (**S4 Table**) in the supplementary materials.

- **Change in Manuscript:**

1. **Split and Renamed Tables:** The original, dense Table 3 has been split into two separate tables:

- **New Table 3 (Main Manuscript):** The main manuscript now contains a new, concise **Table 3** titled: "**Adjusted Hazard Ratio of Hematologic Neoplasm Development for Overall Risk and Key Subgroups, Stratified by Prescription Duration of Hydralazine**". This table presents the "Overall" risk and the data for only the three key subgroups mentioned above.
- **New Supplemental Table (S4 Table):** The data for the remaining nine hematologic neoplasm subgroups have been moved to a new table in the supplementary materials, **Table S4**, titled: "**Adjusted Hazard Ratio for Remaining Hematologic Neoplasm Subgroups, Stratified by Prescription Duration of Hydralazine**".

1. **Restructure the subsection substantially (pages 15–16, lines 334–347):**
   - We replaced the dense paragraphs with a concise summary that highlights the main duration-dependent finding for overall risk.
   - The main manuscript now contains **a new, streamlined Table 3**, which presents the overall risk and the results for the three most robustly significant subgroups.

- **Comment 8:** “HLZ prescription was found to delay HN development” – please note that this statement as currently written implies causality. Please revise.
- **Response:**
- Thank you for this important correction. We agree that the original phrasing "found to delay" implied causality, which is not supported by our observational study design. We have revised this sentence to use non-causal, associative language. Furthermore, we have revised the corresponding subsection heading to ensure consistency and remove any implication of an "effect."
- **Change in Manuscript:**
  1. **Subsection Heading:** The heading for the section has been changed to "**Association of long-term hydralazine use with hematologic neoplasm incidence**". **(page 16, line 367)**
  2. **Sentence Revision:** The sentence now reads: "**Long-term hydralazine use was associated with a longer time to hematologic neoplasm diagnosis**." **(pages 16–17, lines 368–369)**
- **Comment 9:** “Table 4 presents the results of the sensitivity analysis, which were consistent with those presented in Table 3.” – In the 'first year excluded' analysis, the results for ≥180 days are no longer statistically significant. For the "first five years excluded" analysis, the p-value for ≥180 days is .001, meaning it did not reach statistical significance according to your threshold of <.001.
- **Response:**

Thank you for this critical observation. Our original statement was an oversimplification. We have revised this section to describe the results of the sensitivity analysis more accurately.

- **Change in Manuscript:** The text now reads: "**Table 4** presents the results of this analysis. After excluding patients diagnosed within the first year, the association for the overall ≥180 days group was no longer statistically significant (adjusted subdistribution hazard ratio [adjusted sHR] = 0.787, 95% confidence interval [0.646,0.980]; *P* = .03). Similarly, after excluding the first five years, the association for this group did not meet our pre-specified threshold (adjusted sHR = 0.798, 95% CI [0.659,0.950]; *P* = .001). However, the duration-dependent pattern remained robust; the association for the longest-duration subgroup (≥ 668 days) remained statistically significant in both the 1-year exclusion (adjusted sHR = 0.674, 95% CI [0.555,0.838]; *P* < .001) and 5-year exclusion (adjusted sHR = 0.684, 95% CI [0.565,0.858]; *P* < .001) analyses." **(pages 17–18, lines 388–397)**
- **Comment 10:** “S8 Table revealed…” – the table or the analysis? Please revise for clarity.
- **Response:**

Thank you for pointing out this imprecision. You are absolutely correct; a table presents data, but the analysis reveals the findings. We have revised this sentence to improve clarity and accuracy.

- **Change in Manuscript:** The sentence now reads: "**The mortality analysis** showed that there was no significant difference in hematologic neoplasm-related mortality between the exposure group and reference group (adjusted HR = 0.884, 95% confidence interval [0.632,1.238]; *P* = .265, **S9 Table**)" **(page 18, lines 399–402)**
- **Comment 11:** Please provide the unadjusted comparisons as well as the adjusted comparisons in all relevant Tables.
- **Response:**

Thank you for this request. We agree that providing the unadjusted (crude) comparisons alongside the adjusted data is essential for transparency and allows the reader to assess the magnitude and direction of confounding.

We have performed the unadjusted analyses for all relevant tables. To maintain the clarity and readability of the main tables (which are already information-dense), we have placed these unadjusted results in a new, parallel set of tables in the supplementary materials (**S10 Table–S16 Table**). Each new supplemental table directly corresponds to an adjusted table in the main text or supplement, making comparison straightforward. We have also updated the main text to reference these new tables.

- Change in Manuscript
  1. Addition of New Supplemental Tables: We have added **S10 Table–S16 Table** to the supplementary materials. These new tables contain the unadjusted (crude) hazard ratios for the analyses presented in **Table 2, Table 3 & S4 Table, Table 4, S2 Table, S3 Table, S5 Table, and S9 Table**.
  2. References in Main Text: We have added a statement in the "**Statistical analysis**" section to mention this: "**; the corresponding unadjusted (crude) hazard ratios are provided in S10 Table–S16 Table.**" **(page 13, lines 275–278)**
  3. **Titles of New Supplementary Tables**: The new supplementary tables have been titled as follows:
- Table S10. Unadjusted (Crude) Hazard Ratios for Risk Factors Associated with Hematologic Neoplasm Development (corresponds to **Table 2**)
- Table S11. Unadjusted (Crude) Hazard Ratios for Hematologic Neoplasm Development, Stratified by Prescription Duration of Hydralazine (corresponds to **Table 3 and S4 Table**)
- Table S12. Unadjusted (Crude) Hazard Ratios for Sensitivity Analysis of Hematologic Neoplasm Development (corresponds to **Table 4**)
- Table S13. Unadjusted (Crude) Subdistribution Hazard Ratios for First-Event and Multiple-Event Models (corresponds to **S2 Table**)
- Table S14. Unadjusted (Crude) Hazard Ratios for Hematologic Neoplasm Risk Associated with Hypertension (corresponds to **S3 Table**)
- Table S15. Unadjusted (Crude) Subdistribution Hazard Ratios for Leave-One-Out Sensitivity Analysis (corresponds to **S5 Table**)
- Table S16. Unadjusted (Crude) Hazard Ratios for Mortality Analysis (corresponds to **S9** **Table**)
- **Comment 12:** Please specify the variables controlled for in all relevant Tables (pointing to the relevant section is not sufficient).
- **Response:**

Thank you for this essential request. Pointing to the methods section is insufficient for clarity, and we agree that each table reporting adjusted data should explicitly state the variables used for adjustment. We have now added **a detailed footnote** to every relevant table to ensure the analyses are transparent and self-contained.

- **Change in Manuscript:** We have added a comprehensive footnote to all tables presenting multivariable-adjusted data (**Table 2, Table 3, Table 4, S2 Table, S3 Table, S4 Table, S5 Table, and S9 Table**).
  1. This new footnote explicitly lists all variables that were controlled by the models, which included:
     - **Demographics**: sex, age, insured premium, location, urbanization level, and level of hospital.
     - **Comorbidities:** congestive heart failure, pulmonary embolism, gastrointestinal hemorrhage, cerebral thrombosis, ischemic heart disease, vascular insufficiency of intestine, obesity, malignant neoplasm of kidney/renal pelvis, acute glomerulonephritis/nephrotic syndrome, proteinuria, gestational hypertension, and asthma.
     - **Other Variables:** normal pregnancy and Charlson Comorbidity Index_Revised (CCI_R).
     - **Medications:** aspirin, celecoxib, itraconazole, mebendazole, leflunomide, thalidomide, valproate, metformin, auranofin, statins [nystatin, lovastatin, pravastatin, simvastatin, atorvastatin, pitavastatin, rosuvastatin, cilastatin], bisphosphonates [alendronate and risedronate], bromocriptine, chlorprothixene, clotrimazole, quinacrine, ivermectin, verteporfin, clarithromycin, hydroxychloroquine, tofacitinib, gefitinib, curcumin, chlorhexidine, and axitinib.
  2. **The revised footnote is presented as**:

All variables controlled by the models (adjusted hazard ratio and adjusted subdistribution hazard ratio) include demographics (As shown above), comorbidities (As shown above), other variables (As shown above), and medications (As shown above).

**DISCUSSION**

- **Comment 1:** Please remove claims of novelty or primacy (e.g. 'for the first time').
- **Response:**

Thank you for this comment. We have reviewed the manuscript and removed all claims of novelty or primacy, such as "for the first time." We agree that this language is not appropriate for the study.

- **Change in Manuscript:**

1. **Original Sentence:** "This is the **first** nationwide retrospective cohort study to explore the chemopreventive effects of AHAs on HN development in patients with HTN.
2. **Revised Sentence:** "This nationwide retrospective cohort study investigated the association between the use of AHAs and the risk of developing hematologic neoplasms in patients with hypertension." **(page 18, lines 411–413)**

- **Comment 2:** “dose-dependent manner” – Examining different durations is not the same as conducting a dose-response analysis. Please remove these claims throughout the main text.
- **Response:**

Thank you for this crucial correction. You are absolutely right, and we apologize for the imprecise language. Our analysis was stratified by the cumulative duration of prescription, not by different daily dosages. Therefore, referring to the results as "dose-dependent" was incorrect.

- **Change in Manuscript:** we have replaced all instances of "dose-dependent" throughout the entire manuscript
  1. **Abstract (Conclusion)**: The conclusion has been revised to state the association was "**a lower, duration-dependent risk** **of hematologic neoplasms**". **(page 4, line 62)**
  2. **Discussion**: The phrase "in a dose-dependent manner" has been corrected to "**a** **duration-dependent inverse association**".**(page 18, line 417)**
  3. **Discussion**: A later reference to a "dose-dependent relationship" has also been corrected to "**duration-dependent relationship**".**(pages 23–24, lines 543–544)**
- **Comment 3:** “indicating that HLZ may exert favorable effects through several biological regulatory mechanisms.” – How can conclusions about biological regulatory mechanisms be drawn from a retrospective cohort study?
- **Response:**

Thank you for this crucial point. You are absolutely correct; our retrospective, observational study cannot draw conclusions about biological mechanisms. Our intention was not to draw a conclusion, but to speculate on potential explanations for our observed association, which we then support in the following sentences by citing existing in vitro and preclinical literature. The original phrasing ("indicating that HLZ may exert favorable effects") was imprecise and inappropriately implied a direct conclusion.

- **Change in Manuscript:** The sentence has been revised to: " this finding suggests the association may be linked to several biological regulatory mechanisms." **(page 19, line 434–436)**
- **Comment 4:** Please transparently mention whether a study you reference in your discussion was conducted in animals or is based on cell lines.
- **Response:**

Thank you for this excellent point. We agree that for a study discussion to be scientifically rigorous, the context of the cited preclinical data (e.g., animal, cell line, or *in vitro* assay) must be transparently stated. We have carefully reviewed our **Discussion** section and have now explicitly added this context for each of the referenced studies.

- **Change in Manuscript:** We have revised the paragraph in the Discussion section that describes the potential biological mechanisms of hydralazine:
  1. The claim regarding AKT activation (**citation [54]**) is now specified as **an observation from an animal sepsis model**. **(page 20, lines 444–445)**
  2. The finding on **caspase levels** (**citation [55]**) is now clarified as being from **a rat model of cardiac injury**. **(page 20, line 446)**
  3. The inhibition of **glutamic-oxaloacetic transaminase 1** (**citation [56]**) is now identified as a discovery from an ***in vitro* screening assay**. **(page 20, lines 448–449)**
  4. The inhibition of **angiogenesis** (**citation [58]**) is now described as being demonstrated in both ***in vitro* and animal studies**. **(page 20, line 451)**
  5. We have also ensured that our other citations in this section, which were already compliant, remain clear.
     - "in **monocytic leukemia cells**"-(**citation [52]**) **(page 19, lines 439)**
     - "in **human leukemic T cells**"-(**citation [17]**) **(page 19, lines 447–448)**
     - "***in vitro***" for mebendazole- (**citation [43]**) **(page 21, lines 493)**
- **Comment 5:** l.455-464: Please clarify how this is relevant for your study.
- **Response:**
- Thank you for this request for clarification. We included this paragraph to address a critical and well-known clinical consideration regarding hydralazine: the risk of hydralazine-associated lupus-like adverse effects (HAAEs).

While our study focuses on the association between hydralazine use and a lower risk of hematologic neoplasms, any discussion of this finding's potential implications would be incomplete without addressing the drug's primary, well-documented toxicity. This is a major, practical objection that any clinician or researcher would immediately raise.

**The purpose of this paragraph is to contextualize our findings within this known risk profile**. We cite existing literature to show that **the dosages previously associated with a lower risk of hematologic neoplasms in a large cohort are reportedly below the daily dose at which HAAEs typically become a clinical concern**. **This addresses the main safety objection** and helps establish that further investigation into the association we observed is both clinically plausible and warranted.

- **Change in Manuscript:** To improve clarity and fully align with the non-causal standard of our paper, we have revised this paragraph
  1. **Original text**: "...a daily dose of <34 mg exerted significant chemopreventive effects..."
  2. **Revised text:**
     - We have added a topic sentence to the beginning of the paragraph in the Discussion section to explicitly state its relevance: "**A critical consideration for these findings, however, is the known safety profile of long-term hydralazine use.**" **(page 22, lines 503–504)**
     - We also removed the causal language. The text now reads, "...a daily dose of <34 mg **was associated with a significantly lower risk** of overall hematologic neoplasm..." **(page 22, lines 507–508)** and "...suggesting that the dosage (<34 mg per day) **associated with a lower risk** of hematologic neoplasm was considerably below the dosage at which HAAEs have been observed." **(page 22, lines 511–513)**
- **Comment 6:** Due to the retrospective and observational study design, temper any claims regarding clinical use or use in chemoprevention.
- **Response:**

This is a critical point. We acknowledge that our previous **Discussion and Conclusion** over-interpreted our observational findings, especially regarding chemoprevention. We have carefully revised these sections to temper all claims, remove any language suggesting or recommending clinical application, and explicitly re-focus the interpretation on our study's associations.

**We now position the findings as a basis for *future* investigation, not as a support for *current* clinical use**.

- **Change in Manuscript:** The **Discussion and Conclusion** sections have been extensively revised to remove all claims of chemoprevention and clinical use.

1. The opening sentence of the **Discussion** was changed from "explore the chemopreventive effects" to "**investigated the association between the use of AHAs and the risk**...". **(page 18, lines 412–413)**
2. All suggestions of hydralazine as a "protective agent" or having "chemopreventive effects" have been removed and replaced with neutral, associative descriptions (e.g., "**supporting the robustness of the observed association…**"). **(page 24, lines 554–555)**
3. The final Conclusion has been rewritten. It no longer suggests "Adjuvant therapy... may offer chemopreventive effects" and instead states that the "**association suggests... hydralazine use may be linked to a lower incidence... warranting further prospective studies to investigate this relationship**." **(page 25, lines 577–580)**
